# Critical Evaluation of Gamma-Irradiated Serum Used as Feeder in the Culture and Demonstration of Putative Nanobacteria and Calcifying Nanoparticles

**DOI:** 10.1371/journal.pone.0010343

**Published:** 2010-04-26

**Authors:** Jan Martel, Cheng-Yeu Wu, John D. Young

**Affiliations:** 1 Laboratory of Nanomaterials, Chang Gung University, Gueishan, Taoyuan, Taiwan, Republic of China; 2 Department of Biochemistry and Molecular Biology, Graduate Institute of Biomedical Sciences, Chang Gung University, Gueishan, Taoyuan, Taiwan, Republic of China; 3 Research Center of Bacterial Pathogenesis, Chang Gung University, Gueishan, Taoyuan, Taiwan, Republic of China; 4 Laboratory of Cellular Physiology and Immunology, The Rockefeller University, New York, New York, United States of America; 5 Biochemical Engineering Research Center, Mingchi University of Technology, Taishan, Taipei, Taiwan, Republic of China; University of California Merced, United States of America

## Abstract

The culture and demonstration of putative nanobacteria (NB) and calcifying nanoparticles (CNP) from human and animal tissues has relied primarily on the use of a culture supplement consisting of FBS that had been γ-irradiated at a dose of 30 kGy (γ-FBS). The use of γ-FBS is based on the assumption that this sterilized fluid has been rid entirely of any residual NB/CNP, while it continues to promote the slow growth in culture of NB/CNP from human/animal tissues. We show here that γ-irradiation (5–50 kGy) produces extensive dose-dependent serum protein breakdown as demonstrated through UV and visible light spectrophotometry, fluorometry, Fourier-transformed infrared spectroscopy, and gel electrophoresis. Yet, both γ-FBS and γ-irradiated human serum (γ-HS) produce NB/CNP in cell culture conditions that are morphologically and chemically indistinguishable from their normal serum counterparts. Contrary to earlier claims, γ-FBS does not enhance the formation of NB/CNP from several human body fluids (saliva, urine, ascites, and synovial fluid) tested. In the presence of additional precipitating ions, both γ-irradiated serum (FBS and HS) and γ-irradiated proteins (albumin and fetuin-A) retain the inherent dual NB inhibitory and seeding capabilities seen also with their untreated counterparts. By gel electrophoresis, the particles formed from both γ-FBS and γ-HS are seen to have assimilated into their scaffold the same smeared protein profiles found in the γ-irradiated sera. However, their protein compositions as identified by proteomics are virtually identical to those seen with particles formed from untreated serum. Moreover, particles derived from human fluids and cultured in the presence of γ-FBS contain proteins derived from both γ-FBS and the human fluid under investigation—a confusing and unprecedented scenario indicating that these particles harbor proteins from both the host tissue and the FBS used as feeder. Thus, the NB/CNP described in the literature clearly bear hybrid protein compositions belonging to different species. We conclude that there is no basis to justify the use of γ-FBS as a feeder for the growth and demonstration of NB/CNP or any NB-like particles in culture. Moreover, our results call into question the validity of the entire body of literature accumulated to date on NB and CNP.

## Introduction

We have earlier [1]–[5] attempted to dissect the biology of the so-called nanobacteria (NB)—microorganisms that are supposedly exotic, slow-growing, pleomorphic, infectious, pathogenic, sub-micrometer-sized (50–500 nm), and coated with carbonate hydroxyapatite (HAP) [6]–[20]. These anomalous characteristics, considered unprecedented for any microorganism known to date, have been refuted on both epistemological [21] and experimental [1]–[5], [22]–[24] grounds. In our hands, NB are no more than organic-mineral complexes—in fact, they appear as predominantly protein-mineral complexes—that comprise the substrates needed for the normal calcium-carbonate-phosphate homeostasis [1]–[5]. Despite the large body of evidence directly contradicting the claims for NB as living microorganisms, studies implicating NB as infectious agents of disease have continued largely unheeded [25]–[29], with this same association being extended now to the so-called calcifying nanoparticles (CNP), a revised term used to designate NB-like particles by the NB proponents [14]–[20].

Among the more exotic features of the NB biology that has not been addressed to date is the widespread use of fetal bovine serum (FBS) that had been subjected to at least 30 kGy of gamma-radiation as a feeder to support the slow growth of the putative NB in culture [6]–[8], [30]–[38]. The use of gamma-irradiated FBS (γ-FBS) as a growth feeder for NB is based on the initial observation that γ-irradiation at a dose of 30 kGy or higher appeared to completely abolish the ability of serum to initiate NB formation on its own [7], [39], [40]. The finding that irradiation at a dose of 30 kGy exerted a seemingly lethal effect on the NB present in FBS was deemed significant since, according to the NB proponents, all body fluids, including serum, harbor the ability to develop NB when incubated under appropriate cell culture conditions [6]–[8]—observations that have led in turn to claims for a ubiquitous presence for NB in nature [41]–[49]. This lethal effect on NB exerted by radiation was deemed all the more relevant given the remarkable resistance of the said NB to chemical, heat, and even detergent treatments [6], [39], [50]. By presumably disabling DNA and other macromolecules required for life processes, the γ-irradiation effect was also interpreted as supporting the earlier claims for NB as living microorganisms [6]–[8], [15], [39], [40]. Based on this same lethal effect, γ-FBS was also introduced as a “sterilized” feeder to support the growth of NB in culture [7], [39]. In this sense, the γ-irradiation of FBS was deemed to accomplish a thorough sterilization or eradication of all existing and hidden NB activity inherent in the FBS while allowing the same irradiated serum to function as a cell feeder when added to tissue homogenates or fluids harboring the presumptive NB.

Using this logic and in spite of sketchy data, later demonstrations of NB and CNP in human tissues invariably called for their culture in the presence of γ-FBS [6]–[8], [30]–[38]. Table 1 summarizes the primary studies that have used γ-FBS as feeder to implicate NB in human diseases. It can be seen from this table that, in fact, all major studies published to date implicating a role for NB/CNP in human diseases have used γ-FBS as feeder to demonstrate in culture the presence of NB derived from presumably “infected” tissues! All such studies were carried out based on the unproven assumption that γ-FBS contains important nutritional components required for the slow growth and proliferation of NB from human tissues that would not have been expected to grow otherwise. Strangely, γ-FBS is also used now as feeder for CNP despite the fact that the latter are no longer deemed to be living microorganisms and were coined precisely to skirt the controversies generated by the earlier term “nanobacteria” [51]–[53]. In fact, Table 1 includes the more recent work done on CNP calling for an “infectious” and “contagious” model for the nanobacteria-like particles that have been cultivated from tissues in the presence of γ-FBS and that are then used to demonstrate pathogenicity [18], [25], [27], [28], [37], [38], [51]–[53]. Apparently, the use of γ-FBS in the proliferation of both NB and CNP has become the *sine qua non* needed to both demonstrate and implicate a role for NB/CNP in the pathogenesis of various chronic ailments, including atherosclerosis and renal stone formation (Table 1).

**Table 1:** Chronological literature review of studies based on the use of γ-FBS as feeder to culture NB or CNP depicting the source materials used for culture, the main conclusions reached, and the proposed implications of the cited studies on human diseases.

Year	Authors	Source of NB or CNP	Main Findings	Disease Implications	Ref.
1997	Akerman *et al.*	FBS*	NB inoculated into rabbits were found to accumulate in kidneys and to be excreted into urine	Kidney stone formation	[126]
1998	Kajander & Ciftcioglu	FBS and human serum	NB were heralded as new causes of ectopic calcification and kidney stones in humans	Pathological calcification	[8]
1998	Ciftcioglu & Kajander	FBS*	NB were shown to have cytotoxic effects on mouse fibroblasts maintained in culture	Pathological calcification	[30]
1998	Bjorklund *et al.*	FBS	NB showed high resistance to physical agents like UV, microwaves, heat, and drying	Infectious disease	[39]
1999	Ciftcioglu *et al.*	Kidney stones	Kidney stone formation was described as an infectious disease caused by NB infection	Kidney stone formation	[11]
2000	Hjelle *et al.*	Cyst fluid and urine from PKD** patients	NB were found in kidneys, liver, and urine of PKD** patients and were considered as pathogens	PKD	[12]
2002	Ciftcioglu *et al.*	FBS	The growth of NB appeared to be inhibited by several antibiotics, including tetracycline	Pathological calcification	[31]
2004	Miller *et al.*	Calcified heart tissues	NB-like structures were cultured from calcified arteries and were considered as potential pathogens	Vascular calcification	[32]
2005	Wen *et al.*	Bile/serum from cholecystolithiasis patients	NB were found in bile and serum samples of cholecystolithiasis patients	Cholecystolithiasis	[33]
2005	Puskas *et al.*	Atherosclerotic plaques	NB were cultured from most specimens of carotid and atherosclerotic plaques examined	Vascular calcification	[127]
2006	Wang *et al.*	Bile from cholecystolithiasis patients*	Black pigment gallstones were produced by inoculating bile-derived NB into rabbits	Gallstone formation	[34]
2006	Zhou *et al.*	Nasopharyngeal carcinoma cells	NB were cultured from nasopharyngeal cancer cells; NB co-localized with the SPLUNC protein	Nasopharyngeal cancer	[35]
2008	Ciftcioglu *et al.*	Randall's plaques	NB/CNP cultured from Randall's plaque specimens were described as causing this mineral deposit	Kidney stone formation	[18]
2008	Zhou *et al.*	Urine from patients with prostatitis	Type III prostatitis was associated with NB infection and anti-NB treatments were advocated	Type III prostatitis	[36]
2008	Bratos-Perez *et al.*	Calcified aortic heart valves	Self-replicating CNP were cultured and visualized from calcified human aortic valves	Vascular calcification	[37]
2008	Schwartz *et al.*	Calcified heart tissues and kidney stones*	CNP inoculated intravenously into rabbits produced artery injuries and damage to endothelial cells	Arterial injury	[38]
2009	Jones *et al.*	Urinary tract stone	A urinary stone in one astronaut was attributed to the enhanced growth of NB/CNP during space flights	Urinary tract stone formation	[25]
2009	Zhang *et al.*	Urine and semen from infertile male patients	NB infection was seen as a potential cause of testicular microlithiasis and male infertility	Infertility	[27]
2009	Hu *et al.*	Calcified cardiac valves	NB/CNP were cultured from calcified cardiac valves of patients with rheumatic heart disease	Vascular calcification	[28]
2009	Schwartz *et al.*	Calcified aneurysms	Formation of biofilms by human-derived nanoparticles was reduced by antibiotics, but not RNase	Vascular calcification	[51]
2009	Miller *et al.*	Calcified aneurysms, kidney stones, and FBS*	Biologic nanoparticles of human/bovine origin or HAP crystals inhibited platelet aggregation	Response to arterial injury	[52]
2009	Schwartz *et al.*	Calcified heart tissues and FBS*	Planktonic or floating forms of mammalian-derived nanoparticles in solution produced artery injuries in rabbits	Arterial injury	[53]
2009	Franiczek *et al.*	Carotid artery plaques and kidney stones	CNP were considered as non-living particles with possible implications in ectopic calcification	Pathological calcification	[128]

*In these studies, NB/CNP specimens were first prepared by incubating either human or animal tissues along with γ-FBS. The NB/CNP samples were then tested by inoculation in laboratory animals or through functional assays in cell culture, involving animal or cell species different from the one used to derive the initial NB/CNP isolate.

**PKD: polycystic kidney disease.

The study reported here was designed to assess the role of γ-FBS as a feeder for NB as well as NB-like particles (abbreviated in this paper as NLP). Our results demonstrate that the use of γ-FBS not only suffers from the lack of any evidentiary support that can justify its use as a feeder for the growth of NB in culture but its use is in itself problematic in that it predisposes the carry-over into the NB scaffold of organic components of different species (human tissue and bovine serum constituents) resulting in largely uninterpretable data. Based on our results it would appear that all earlier published studies using γ-FBS to demonstrate and implicate NB and CNP in human diseased tissues are fundamentally flawed.

## Results and Discussion

### Visual Inspection and Turbidity Readings of γ-Irradiated Serum

To evaluate the role of γ-irradiation on NB formation, we irradiated both commercial FBS and human serum (HS) obtained from healthy individuals at six doses ranging from 5 to 50 kGy. We then monitored the ability of these solutions to produce the so-called NB and NLP in culture according to established procedures [6]–[8]. In this manuscript, we have used the two terms NB and NLP interchangeably, with the term NB more frequently used to designate particles that are formed slowly in metastable cultures without the addition of precipitating ions, in line with its usage by the original NB proponents [6]–[8], [11]–[13]. On the other hand, the term NLP is used in situations where precipitating ions are present or when the particles are derived through shorter-term cultures, in line with our earlier studies [1]–[5]. At times, when our procedure is supported by both the initial NB literature and the later NLP work performed by our and other laboratories, we have also used the combined term “NB/NLP” to broaden its scope of representation. It should be noted that our term NLP is also being used generically with the intent to encompass all other terminologies used earlier for similar particles, including nanoforms [54], CNP [14]–[20], [25]–[29] and “nanons” [24].

**Figure 1:**
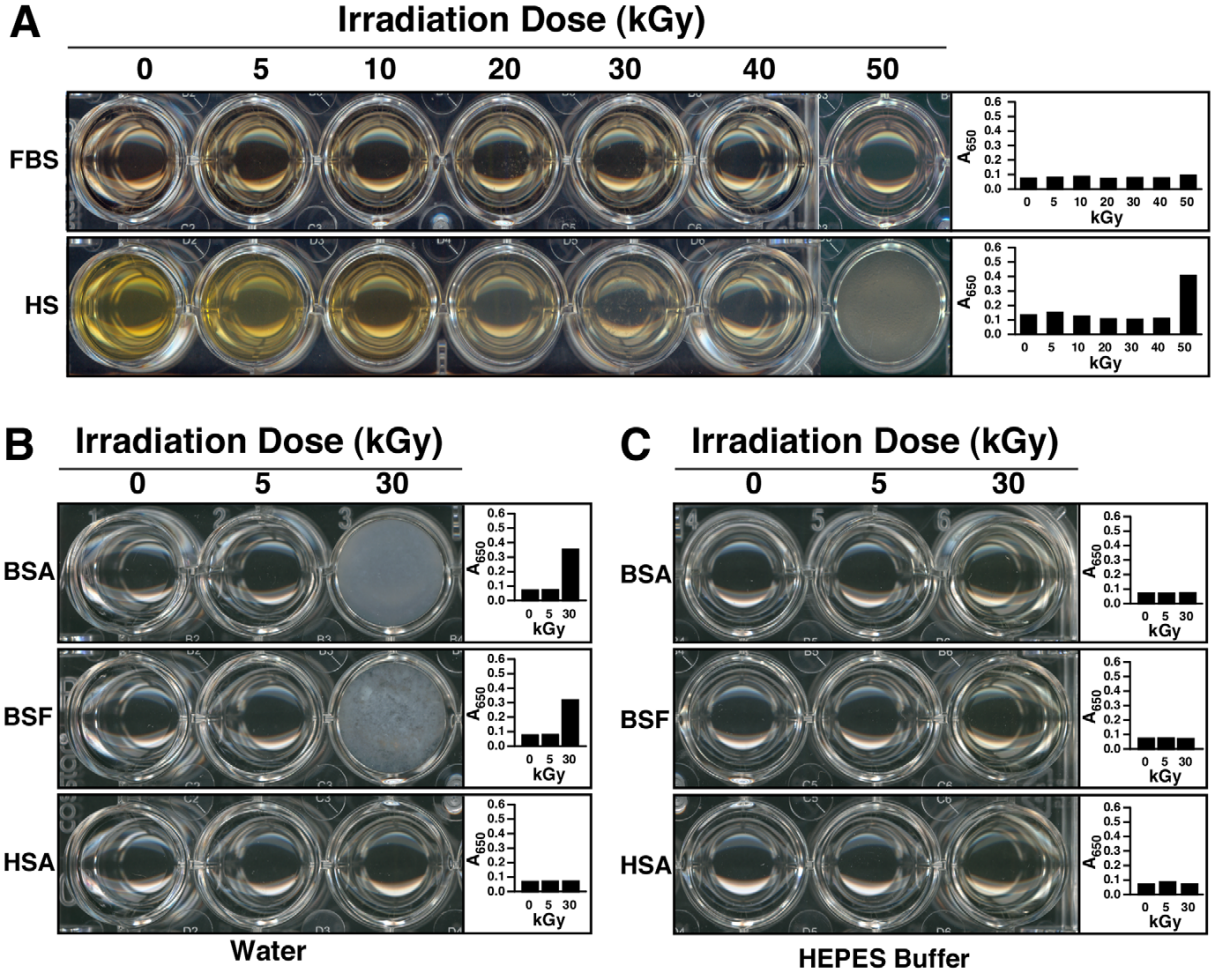
Visual inspection and turbidity readings of γ-irradiated serum and γ-irradiated protein solutions. (A) FBS and HS were γ-irradiated with radioactive cobalt-60 at doses ranging from 5 to 50 kGy, and aliquots of the irradiated solutions were transferred to 24-well plates for visual inspection. Control, non-irradiated FBS/HS were shown for comparison (“0 kGy”). Turbidity readings were performed at 650 nm as shown on the right. γ-Irradiation produced a dose-dependent discoloration of both sera. In addition, high doses of irradiation induced a precipitation reaction, as seen especially in the case of 50-kGy-irradiated HS. Solutions of BSA, BSF, and HSA prepared in either (B) water or (C) HEPES buffer were γ-irradiated at either 5 or 30 kGy, and were processed the same way. While γ-irradiation of both BSA and BSF prepared in water produced extensive precipitation at 30 kGy, no precipitation was detected for the other protein solutions.

In order to first examine the effect of γ-irradiation on serum, we transferred the irradiated solutions into 24-well plates for visual inspection and turbidity readings using spectrophotometry at 650 nm (A_650_). Through visual inspection of γ-FBS irradiated at a dose of 5 to 40 kGy, we observed a slight discoloration that appeared to be γ-dose dependent, with the 50-kGy sample appearing largely discolored (Fig 1A, FBS row, compare with the untreated control labeled as “0 kGy”). Moreover, γ-FBS irradiated at 50 kGy became slightly turbid and showed a small amount of white precipitate in solution (Fig. 1A; note the small increase in A_650_ depicted in the graph on the right). For HS, we noticed that γ-irradiation produced a more pronounced discoloration as compared with FBS (Fig. 1A). Moreover, 50 kGy produced a much higher turbidity in HS than FBS, which could be seen also from the A_650_ readings (Fig. 1A, right). The increase of turbidity seen with γ-HS was accompanied by the accumulation of small amounts of white particulate matter, a phenomenon that was more readily noticeable at doses of 20 kGy and higher (Fig. 1A, HS row). Notably, γ-irradiation of HS at 50 kGy produced extensive white precipitation throughout the serum solution which also became more viscous as seen through pipetting (Fig. 1A, HS row, well marked as “50 kGy”).

**Figure 2:**
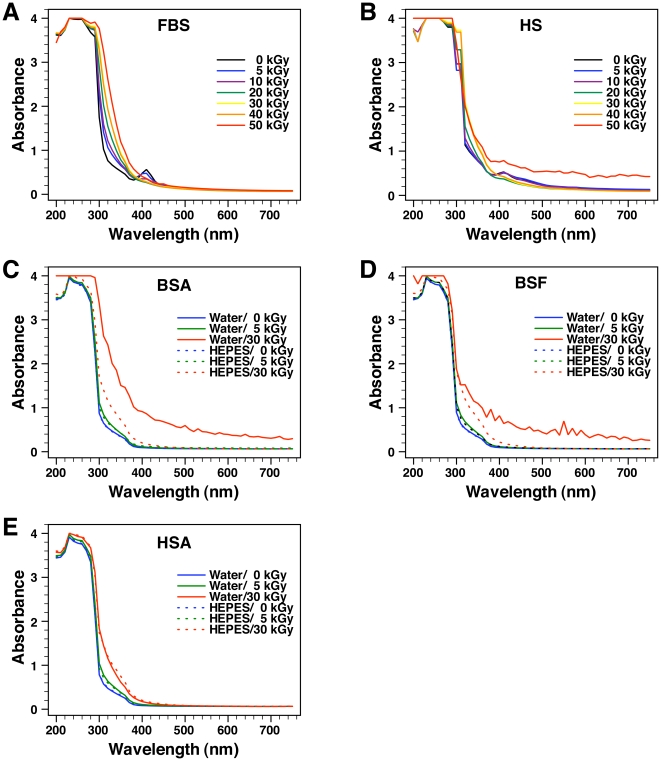
Light absorbance of γ-irradiated serum and γ-irradiated proteins solutions monitored in continuous-scanning mode. Absorbance of (A) FBS and (B) HS and of the protein solutions containing (C) BSA, (D) BSF, and (E) HSA prepared in either water (solid line) or HEPES buffer (dashed line) was monitored from 200 to 750 nm before and after γ-irradiation at the dose indicated.

To confirm the changes observed by visual inspection, we monitored light absorbance of the serum solutions using spectrophotometry in continuous-scan mode. Absorbance was examined at wavelengths ranging from 200 to 750 nm, which cover part of the ultraviolet (UV) spectrum (10–380 nm) and the entire visible spectrum (380–750 nm). Absorbance at 260 nm is routinely used to detect nucleic acids (i.e. DNA and RNA), while absorbance at 280 nm is mainly attributed to the aromatic amino acids found in proteins [55]. On the other hand, absorbance above 330 nm can also be attributed to light scattering and, in the case of serum, it may indicate the presence of particulate matter in solution [55]. As can be seen from Figure 2A, the absorbance of both non-irradiated (untreated) FBS and γ-FBS peaked between 230 and 270 nm. The high absorbance seen in this region can be mainly attributed to the presence of high amounts of proteins in the serum, but nucleic acids probably also contribute to forming this peak [55]. At wavelengths between 300 and 380 nm, we noticed that the absorbance associated with γ-FBS gradually increased with the dose of irradiation used. That is, there was a gradual dose-dependent shift of the absorbance peak to the right, reflecting probably a major change in serum composition (Fig. 2A). On the other hand, the increased absorbance at these wavelengths could not be explained by a turbidity increase which would have been visible to the eyes or through the A_650_ readings as shown in Figure 1A. To the right, the absorbance peak seen at 415 nm for non-irradiated FBS gradually decreased with γ-irradiation at doses between 5 to 20 kGy and largely disappeared with higher doses (Fig. 2A). Absorbance at this wavelength is usually associated with the heme group found in hemoglobin [56], [57]. Finally, at wavelengths above 440 nm, we observed no significant changes in absorbance between control, untreated FBS and the γ-FBS solutions that had been irradiated at 5 to 50 kGy (Fig. 2A).

For non-irradiated HS and γ-HS, the absorbance observed through continuous scanning also peaked between 230 and 270 nm, reflecting mainly the presence of proteins in the serum (Fig. 2B). At wavelengths between 320 and 360 nm, a progressive absorbance increase was noted for γ-HS irradiated at 20 to 50 kGy (Fig. 2B). That is, as seen earlier with γ-FBS, there was a noticeable shift of the absorbance curve to the right that was γ-dose-dependent. As for the heme peak at 415 nm associated with HS, the absorbance was also similarly decreased with increasing radiation doses between 5 to 20 kGy (Fig. 2B). On the other hand, the absorbance at 415 nm for γ-HS irradiated at 30 to 50 kGy was higher than the 20-kGy sample, appearing to result from the rightward shift in the absorbance curve (Fig. 2B). In this case, the higher absorbance of γ-HS irradiated at 30 to 50 kGy may possibly also reflect the presence of particulate matter in these solutions as seen earlier in Figure 1A. At wavelengths above 380 nm, we observed that the absorbance of γ-HS irradiated at 50 kGy was considerably higher than control non-irradiated HS (Fig. 2B). This absorbance increase in the γ-HS solution irradiated at 50 kGy was consistent with the abundant white precipitation observed by visual inspection and by A_650_ reading (Fig. 1A). On the other hand, no substantial turbidity increase was noted above 380 nm for the other γ-HS solutions (Fig. 2B), an observation that was again consistent with both the visuals and the A_650_ readings shown in Figure 1A.

These changes in serum produced by γ-irradiation can be attributed to the well-known damaging effects of γ-rays on biological molecules. γ-Rays, consisting of electromagnetic radiation of high energy (i.e. above 100 keV), are known to be able to break covalent bonds in any macromolecule present in a biological fluid [58], [59]. While most biological molecules can be damaged directly by γ-rays, the major part of the damage caused by this treatment is usually attributed to the free radicals hydroxyl (·OH) and superoxide (O_2_^−^) produced during the hydrolysis of water [58]–[60]. These highly reactive free radicals not only can break covalent bonds, but they are also known to alter the functional groups of biological molecules by mediating oxidation, peroxidation, de-amination, and decarboxylation reactions, among others [58]–[60]. In turn, the ionization and alteration produced this way can lead to the degradation of biological molecules or can induce their denaturation, aggregation, or precipitation.

In this sense, the deleterious effects of γ-irradiation can be illustrated by the dose-dependent discoloration of serum (Fig. 1A). The yellow color of serum is usually attributed to the presence of bilirubin, a heme degradation product [61], [62]. The gradual discoloration of serum can thus be interpreted as a dose-dependent breakdown of bilirubin by γ-rays, which had indeed been documented by other groups [62], [63]. The damaging effects on biological molecules produced by γ-irradiation can also be illustrated by the irradiation-induced decrease of the heme-related absorbance peak at 415 nm seen in Figures 2A and B, suggesting that γ-irradiation may have caused a γ-dose-dependent destruction of the heme group found in hemoglobin. In this respect, it should be noted that the reactive oxygen species produced by γ-irradiation are known to target proteins [64] which are present in relatively high amounts in serum. As such, it is possible that the precipitation seen in serum after treatments with high doses of γ-irradiation, like 50 kGy, may be partially due to the precipitation of serum proteins. In line with this possibility, the higher propensity for HS to precipitate compared to FBS could be attributed to the earlier observation that the concentration of proteins in HS is almost twice that of FBS, with our lots of HS containing on average 60 mg/ml versus 32 mg/ml for FBS [4]. However, we cannot rule out the participation of other components like lipids and nucleic acids in the precipitation and turbidity increase associated with the γ-irradiated serum solutions.

Given that γ-irradiation of FBS did not produce visible changes at a dose of 30 kGy, it is possible that the effects of irradiation were overlooked earlier when this γ-FBS sample was established as a supplement for the culture of NB [6]–[8]. On the other hand, it appears that γ-irradiation produced several irreversible changes in the composition of serum, as seen above with the breakdown of bilirubin and heme and the absorbance changes. These changes could in turn affect the ability of this biological fluid to form NB and NLP in culture, a possibility that we seek to address in more detail in later sections.

### Visual and Turbidity Changes Associated with γ-Irradiated Albumin and Fetuin-A

We had earlier observed that serum proteins play an important role in the formation of NB and NLP, presumably through a dual inhibition-seeding mechanism [2]–[5]. NB/NLP derived from both HS and FBS were shown to be enriched for albumin, complement components 3 and 4A, apolipoproteins A1 and B100, fetuin-A, and a number of other calcium- and apatite-binding proteins [4]. Of the proteins identified as part of the NB/NLP scaffold, we studied in greater detail the roles of albumin and fetuin-A, which also represent the main calcium and apatite-binding proteins in the serum [2]–[4]. Both were shown to interact actively with calcium and phosphate resulting in the formation of particles, spindles, and films [2], [4].

In order to further verify the possibility that γ-irradiation abolishes NB/NLP formation by targeting proteins, we first irradiated solutions of purified albumin and fetuin-A with γ-rays and then processed them for visual inspection and spectrophotometry exactly as described above. For these studies, we irradiated commercially available albumin and fetuin-A proteins at either 5 or 30 kGy. Bovine serum albumin (BSA), bovine serum fetuin-A (BSF), and human serum albumin (HSA) were used for our studies since these proteins were readily available in large quantities. The lyophilized form of each protein was thus dissolved at a concentration of 10 mg/ml in either double-distilled water (hereafter abbreviated as “water”) or a HEPES buffer with an ionic composition, osmolarity, and pH similar to those of physiological fluids (see *Materials and Methods*). To prevent contamination, all solutions were sterilized by filtration through a 0.2-µm membrane prior to γ-irradiation and they were processed under sterile cell culture conditions at all time.

Dissolution and homogenization of the proteins in water produced clear, translucid solutions (Fig. 1B, columns depicted as “0 kGy”). Following γ-irradiation at 5 kGy, both BSA and BSF dissolved in water remained translucid (Fig. 1B). On the other hand, irradiation with 30 kGy produced an abundant white precipitate (Fig. 1B). Precipitation in these solutions was also confirmed by an increase of A_650_ readings (Fig. 1B, graphs on the right). In contrast to the bovine proteins, the HSA solution prepared in water remained translucid even after γ-irradiation at 30 kGy (Fig. 1B, both visuals and A_650_ readings on the right). While it is likely that doses of γ-irradiation above 30 kGy would have produced changes in this protein solution, we used treatments up to this dose to be in line with the earlier NB studies [6]–[8], [30]–[38].

Dissolution of the same proteins (BSA, BSF, and HSA) in HEPES buffer also produced clear homogeneous solutions (Fig. 1C). However, in contrast to the solutions prepared in water, no precipitation was noticed when either one of these three protein solutions prepared in HEPES buffer were γ-irradiated up to 30 kGy, as seen through both visuals and A_650_ readings (Fig. 1C).

To further confirm these observations, we monitored the light absorbance of each protein solution in continuous-scanning mode (Fig. 2C–E). We first noticed that the absorbance of each of these protein solutions peaked near 230 nm (Fig. 2C–E), comparable to the absorbance spectra obtained for the whole serum solutions, which also peaked at 230–270 nm (Fig. 2A and B). Little or no change in absorbance was detected in the protein solutions irradiated at 5 kGy (Fig. 2C–E), in line with the absence of precipitates seen earlier (Fig. 1B and C). As for the irradiation of the protein solutions with 30 kGy, the following results were observed. In the case of BSA and BSF, changes in the absorbance spectra were more pronounced with the protein solutions prepared in water, with marked increases in absorbance seen throughout the visible spectrum scanned (Fig. 2C and D). These changes were in line with the precipitation and the increased turbidity seen earlier (Fig. 1B). On the other hand, the HEPES solutions of these same two proteins produced a much more subdued increase in absorbance in the range between 300 to 460 nm, an increase that was nonetheless significant when compared to their respective non-irradiated control (Fig. 2C and D). As for the HSA irradiated with 30 kGy, a low increase of absorbance was noticed between 300 and 460 nm with the protein dissolved in either water or HEPES buffer (Fig. 2E).

These observations indicated that γ-irradiation at 30 kGy was sufficient to induce the precipitation of BSA and BSF prepared in water, but not of HSA prepared under similar conditions (Fig. 1B). On the other hand, the HEPES buffer appeared to confer stability to these same protein solutions, preventing the precipitation of either BSA or BSF after γ-irradiation with 30 kGy (Fig. 1C). Nonetheless, at this irradiation dose, conformational changes in all three proteins were detected, as reflected through the absorbance increases seen in the visible range spanning between 300 and 400 nm (Fig. 2C–E). Thus, while the HEPES buffer may have acted as a potent scavenger of free radicals in solution, in line with earlier reports [65], [66], it was obvious that the protection conferred by HEPES against the radiation-induced ionization was not complete. Presumably, the vast repertoire of ionic and organic compounds present in the serum may confer similar protection against the damaging effects of γ-irradiation. In fact, biological substrates like uric acid and bilirubin, and amino acids like cysteine are all known to neutralize free radicals and protect the organism against oxidative stress [67], [68]. This same biological protection may have accounted for the relatively high resistance of serum to γ-irradiation, with FBS showing higher tolerance than HS insofar as both spectral changes and precipitation are concerned.

It should be noted that proteins like albumin and fetuin-A represent major protein species found in the serum. For instance, albumin is present at a concentration averaging 23 mg/ml in FBS [69] while it is found in even higher quantities in HS, at 35–45 mg/ml [70]. On the other hand fetuin-A is found in much higher amounts in FBS, at 10–21 mg/ml [71], against 0.7–0.8 mg/ml found in HS [72]. These concentrations are significant given the total protein concentrations of 32 mg/ml found for FBS and 60 mg/ml for HS [4]. Since the visual and spectral changes seen with both albumin and fetuin-A correspond largely to those seen with whole serum (Figs. 1 and 2), the results presented here suggest that the γ-irradiation-induced changes observed with whole serum appear to represent largely the summation of changes seen at the level of individual serum proteins. This inference is all the more relevant to our understanding the role of γ-irradiation in the NB/NLP biology given the fact that serum proteins are an important part of the scaffold of NB/NLP that had been derived from serum [2]–[5].

### Conformational Changes and Unfolding of Serum Proteins following γ-Irradiation as Revealed by Protein Fluorometry

**Figure 3:**
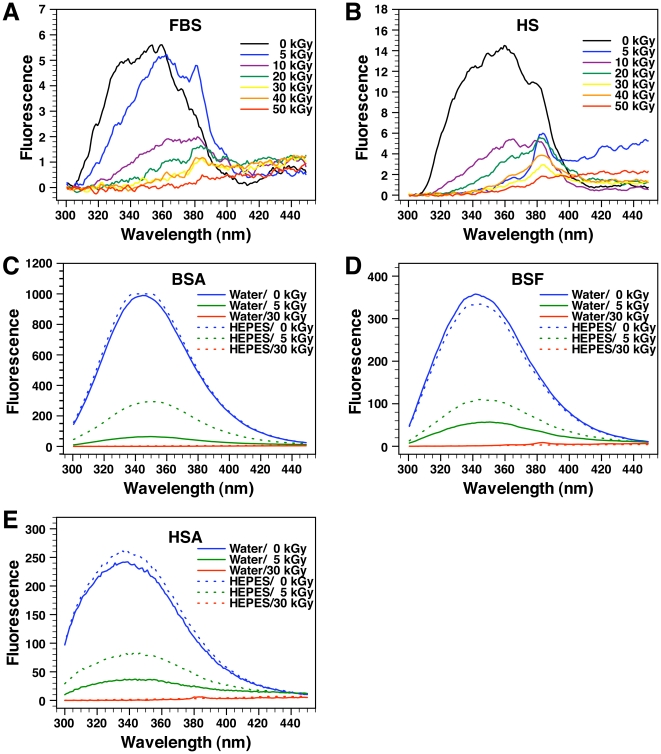
Conformational changes of serum proteins following γ-irradiation as revealed by protein fluorometry. Aliquots of (A) FBS and (B) HS γ-irradiated at the doses indicated were excited with UV light at 280 nm, and the resulting fluorescence emission was monitored between 300 to 450 nm. Control, non-irradiated FBS/HS were included for comparison. Fluorescence was also monitored for both non-irradiated and γ-irradiated solutions of (C) BSA, (D) BSF, and (E) HSA prepared in either water (solid line) or HEPES buffer (dashed line). In most samples, γ-irradiation produced a dose-dependent decrease of fluorescence intensity and a shift of maximum fluorescence to longer wavelengths, consistent with protein unfolding and conformational changes.

To further evaluate the effects of γ-rays on the conformation of serum proteins, we monitored the fluorescence of the serum and protein solutions following γ-irradiation. The serum and protein solutions were first excited with UV light at 280 nm. The resulting fluorescence, attributed mainly to aromatic amino acids, i.e. tryptophan, tyrosine, and phenylalanine [73], [74], was monitored from 300 to 450 nm. For both FBS and HS, γ-irradiation was shown to produce a dose-dependent decrease of fluorescence intensity (Fig. 3A and B). γ-Irradiation resulted also in a marked dose-dependent shift of maximum fluorescence (λ_max_) toward the right, i.e. toward longer wavelengths, for both FBS and HS (Fig. 3A and B). For ease of comparison, these same λ_max_ values obtained in response to various γ-irradiation doses can also be seen depicted in Table 2. For example, in the case of FBS, λ_max_ values of 352 and 359 nm were observed for control, non-irradiated FBS, while the λ_max_ values observed for γ-FBS gradually increased with the dose of irradiation, peaking at 385 nm with 30 and 40 kGy, and 450 nm with 50 kGy (Fig. 3A and Table 2). For HS, a comparable shift of λ_max_ was observed, with 358 nm obtained for control vs. 384 nm and 435 nm with 30 kGy and 50 kGy, respectively (Fig. 3B and Table 2).

**Table 2:** Peak maximum fluorescence (λ_max_) for solutions of FBS and HS before and after γ-irradiation.

	Fluorescence Peak (nm) after Irradiation at the Dose Indicated*						
	0 kGy	5 kGy	10 kGy	20 kGy	30 kGy	40 kGy	50 kGy
**FBS**	352, 359	363	381	384	385	385	450
**HS**	358	385	365	381	384	382	435

*Each λ_max_ value corresponds to the wavelength(s) at which maximum fluorescence was observed in Figure 3.

We also studied the fluorescence emitted by the purified serum proteins that had been excited under the same conditions. In this case, the level of maximum fluorescence observed for control, non-irradiated proteins was much higher than that of non-irradiated serum (Fig. 3C–E, as compared with Fig. 3A–B). For example, the maximum fluorescence of BSA prepared in HEPES buffer reached 1,000 absorbance units while the fluorescence of FBS reached only 5.6 units. HSA in HEPES buffer gave a fluorescent peak of 261 units while HS reached only 14.4 units. The higher fluorescence intensities seen for the sera compared to the serum proteins could not be attributed to their respective protein concentrations since the sera contained on average 3 to 6 times more proteins than the protein solutions (32 mg/ml for FBS and 60 mg/ml for HS vs. 10 mg/ml for each protein solution; see ref. [4]). Instead, the difference of fluorescence intensity observed in this case may very well reflect the complexity of the serum that could have interfered with both UV light absorbance and fluorescence emission. The λ_max_ values obtained for the three non-irradiated protein solutions of BSA, BSF, and HSA, did not appear to be influenced significantly by the nature of the solvent used (i.e. water or HEPES buffer; see Fig. 3C–E and Table 3). In addition, the λ_max_ values obtained for these three proteins were comparable to the λ_max_ values reported earlier in the literature, with 344 nm having been recorded for BSA [75], 340 nm for BSF [76], and 342 nm for HSA [75]. These reported data provide an independent verification of the methodology used here.

**Table 3:** Peak maximum fluorescence (λ_max_) for the protein solutions of BSA, BSF, and HSA before and after γ-irradiation.

	Peak Fluorescence (nm) after Irradiation at the Dose Indicated		
	0 kGy	5 kGy	30 kGy
**BSA/Water**	345	349	**–** *
**BSF/Water**	342	349	384
**HSA/Water**	339	344	384
**BSA/HEPES**	344	351	**–** *
**BSF/HEPES**	343	345	–*
**HSA/HEPES**	338	344	**–** *

The λ_max_ values correspond to the wavelength(s) at which maximum fluorescence was observed in Figure 3.

*Irradiation at 30 kGy completely abrogated fluorescence in some protein solutions resulting in no peak being detected.

Comparable to what had been seen with the whole serum, γ-irradiation produced a marked dose-dependent decrease of fluorescence for all three proteins BSA, BSF, and HSA dissolved in water (Fig. 3C–E). By comparison, a similar, albeit less pronounced, dose-dependent decrease of fluorescence was also observed for the three proteins dissolved in the HEPES buffer (Fig. 3C–E). γ-Irradiation also produced a dose-dependent shift of λ_max_ values for all these proteins prepared in either water or HEPES buffer (Fig. 3C–E and Table 3). It should be noted however that for several protein solutions that had been irradiated with 30 kGy, especially the ones dissolved in HEPES buffer, the absorbance peak was completely abolished and consequently there was no detectable λ_max_ (Fig. 3C–E and Table 3).

It is generally assumed that variations of fluorescence intensity for a protein sample correspond to conformational changes mainly in the context of aromatic amino acids [73], [74]. Moreover, the shift of λ_max_ values toward longer wavelengths is usually considered a sign of protein unfolding [73], [74]. That is, aromatic amino acids present in proteins have been found to emit fluorescence at longer wavelengths when they become exposed to water in solution [73], [74]. Given that aromatic amino acids are buried inside proteins in the native state due to their hydrophobic character, a shift of maximum fluorescence to longer wavelengths can be seen as both water exposure and protein unfolding [74]. Our observations are thus clearly indicative of both conformational changes and unfolding associated with serum proteins that are presumably induced by γ-irradiation in a dose-dependent manner. These conformational changes are much more noticeable with the proteins dissolved in water than in buffer solutions, again indicating that buffers like HEPES and presumably serum are able to confer significant protection against the ionization effects produced by γ-irradiation.

### Changes in the Secondary Structures of Serum Proteins Produced by γ-Irradiation as Revealed by Fourier-Transformed Infrared Spectroscopy

**Figure 4:**
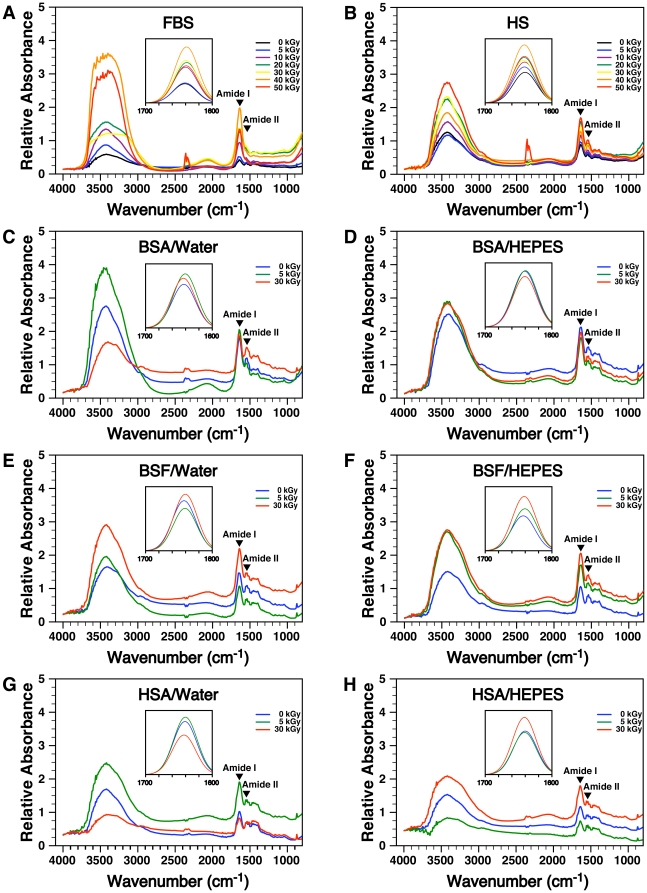
Changes in the secondary structures of serum proteins produced by γ-irradiation as shown by Fourier-transformed infrared spectroscopy. Aliquots of (A) FBS and (B) HS γ-irradiated at the doses indicated were processed for FTIR analysis. The corresponding, non-irradiated sera were also included for comparison. The analysis was also performed for solutions of (C and D) BSA, (E and F) BSF, and (G and H) HSA, all prepared at 10 mg/ml and γ-irradiated at the doses indicated. The protein solutions were prepared in either (C, E, and G) water or (D, F, and H) HEPES buffer. In order to compare the intensities of the major peak at 3,400 cm^−1^ seen with the various irradiation doses, the baseline of each spectrum was aligned at 4,000 cm^−1^. To evaluate the changes seen with the amide-I peak after γ-irradiation, we then normalized the spectra from 1,700 to 1,600 cm^−1^ using a Gaussian-Lorentzian function (insets) as described in the *Materials and Methods*. See text for explanation and interpretation.

To verify the extent of conformational change of serum proteins following γ-irradiation, we relied on Fourier-transformed infrared spectroscopy (FTIR), which is widely used to quantify and characterize the secondary structures of proteins (e.g. α-helices, β-sheets, β-turns, and so forth; see refs. [77], [78]). The FTIR absorbance spectra of γ-irradiated serum solutions were recorded at frequencies ranging from 4,000 to 800 cm^−1^ (Fig. 4A and B). The peak of amide I (1,695–1,610 cm^−1^) is often viewed as the most useful component of an FTIR spectrum for monitoring protein secondary structures [77]–[80]. This peak corresponds mainly to C = O stretching vibrations of the amide group which are particularly sensitive to the formation of hydrogen bonds in the backbone of proteins [77], [78]. In turn, the importance of the amide-I peak in monitoring secondary structures comes from the observation that hydrogen bonds formed in the backbone of proteins are implicated in the formation of the major secondary structures [77], [78]. In comparison, the amide-II peak (1,550 cm^−1^), which arises from a combination of N–H bending (60%) and C–N stretching (40%) of amide bonds, is less influenced by changes in hydrogen bonding, and is therefore considered to have a lower value in determining the secondary structure of proteins [77], [78]. In addition to these peaks, there is also a major band usually associated with amide A (3,300 cm^−1^) and amide B (3,070 cm^−1^) peaks with both representing stretching vibrations seen with the N–H component of amide bonds [78]. However, the bands of amide A and amide B do not appear to be consistently associated with changes in the secondary structures of proteins [78].

When we examined the FTIR spectra of γ-FBS shown in Figure 4A, we first noticed that the peak at 3,400 cm^−1^ showed large variation of intensity following γ-irradiation. To compare the intensity of the peak seen at 3,400 cm^−1^ between samples, the spectra were first manually aligned in order for them to have the same absorbance intensity at 4,000 cm^−1^ (Fig. 4A). With this alignment, we observed that the intensity of the peak seen at 3,400 cm^−1^ usually increased following irradiation (Fig. 4A). The analysis of this band is complicated by the fact that it is found in a spectral region associated with several other common absorbance bands, including those of water [78]. It thus remains unclear whether the variation of absorbance intensity seen in this region correlated with changes in protein secondary structures following γ-irradiation, as also noted in a previous study [78].

In the case of the amide-II peak near 1,550 cm^−1^, we also observed no consistent variation for the γ-FBS samples after aligning the spectra with the baseline at 900 cm^−1^ for comparison (data not shown).

We focused our attention instead on the peak of amide I observed between 1,695 and 1,610 cm^−1^ (Fig. 4A). To compare the relative intensity of this peak between samples and to remove the noise in the spectra obtained, the FTIR spectra were normalized between 1,700 and 1,600 cm^−1^ using a curve-fitting, Gaussian-Lorentzian function (see *Materials and Methods* as well as refs. [78], [79] for more detailed explanation) and a close-up was made on the resulting peaks in order to better depict the subtle changes that occurred in this region (Fig. 4A, inset). We first observed that the resulting amide-I peak in FBS increased in intensity after γ-irradiation, but the increase observed did not seem to always correlate with the dose of radiation used (Fig. 4A, inset). More importantly, following γ-irradiation, we noticed a shift of the amide-I peak toward the right, i.e. toward lower frequencies (Fig. 4A, inset). A shift of the amide-I peak in this context is usually attributed to changes of protein secondary structures [77]–[79]. In order to confirm this possibility and to quantify the level of changes observed, we applied Fourier self-deconvolution (FSD) analysis of the amide-I peaks and estimated the level of secondary structures present in each sample (see *Materials and Methods* and refs. [78], [79]). At the core of this analysis is the presumption that the large peak of amide I represents a combination of several smaller peaks, with each one corresponding in turn to a specific secondary structure such as α-helix, β-sheet, β-turn, and random coil [77]–[80]. In addition, the content of each secondary structure in a protein sample can be quantified by measuring the area under the curve for each peak [77]–[80]. Accordingly, Table 4 shows the level of α-helices, β-sheets, β-turns, and random coils obtained based on this FSD analysis.

**Table 4:** Secondary structures of FBS and HS proteins before and after γ-irradiation as determined by FTIR peak deconvolution analysis of the amide-I band.

		Percentage of Secondary Structure of Serum Proteins after Irradiation at the Dose Indicated						
		0 kGy	5 kGy	10 kGy	20 kGy	30 kGy	40 kGy	50 kGy
**FBS**	**α-Helix**	29.3	19.8	21.2	1.1	5.2	6.9	0.3
	**β-Sheet**	38.5	53.3	66.5	28.1	23.8	34.8	20.9
	**β-Turn**	17.7	10.4	9.3	12.4	17.4	7.4	13.7
	**Random Coil**	14.5	16.5	3.0	58.4	34.2	50.9	65.3
**HS**	**α-Helix**	22.5	36.5	8.5	21.1	27.1	26.7	0.2
	**β-Sheet**	16.8	41.1	16.7	22.2	55.3	25.9	20.9
	**β-Turn**	13.1	11.4	10.1	24.4	11.1	28.5	10.9
	**Random Coil**	47.6	11.7	64.7	32.3	6.5	18.9	68.0

The deconvolution analysis of amide-I peaks was performed using the 4^th^ derivative of the Gaussian-Laurentzian-normalized FTIR spectra as described in the *Materials and Methods*.

We first observed that the content of α-helices, β-sheets, and β-turns of FBS proteins generally decreased after γ-irradiation (Table 4). On the other hand, the content of disordered, random coils increased in most FBS samples following γ-irradiation (Table 4), supporting the idea that this treatment altered the secondary structures of FBS proteins. It should be noted that this trend was not followed however by every sample (e.g. FBS irradiated with either 5 or 10 kGy, which gave erratic readings) and the amounts of any given secondary structure observed did not always vary in a dose-dependent manner with respect to the dose of radiation used (Table 4). Since this particular set of measurements was performed only once, the data given here beckon for caution of interpretation. Nonetheless, when analyzed together, the numbers do support a general trend of change in secondary structure as a function of the γ-dose used.

In the case of HS, the intensity of the peak near 3,400 cm^−1^ also increased drastically with γ-irradiation (Fig. 4B), but the variations observed did not appear to correlate strictly with the dose of radiation used. Concerning the amide-II peak and after aligning the spectra at 900 cm^−1^ for comparison, there was also a general increase in absorbance with γ-irradiation but, again, this increase did not correlate strictly with the level of γ-dose used (Fig. 4B). Likewise, the intensity of the amide-I peak in HS samples was found, with some exceptions, to increase generally in intensity following γ-irradiation (Fig. 4B, inset). We also observed a slight shift of the amide-I peak toward higher frequencies after γ-irradiation of HS (Fig. 4B, inset). Moreover, when we performed the same FSD analysis of the amide-I peaks in these γ-HS samples, we observed changes in the secondary structures of these samples (Table 4). While the changes observed appeared to be more erratic than the results obtained for γ-FBS, they may again reflect a major derangement of native protein conformation in γ-HS samples.

We next repeated the FTIR analysis with the pure proteins solutions described in the previous sections. As seen from the spectra depicted in Figure 4C–H, we observed marked variations in the intensity of the peak at 3,400 cm^−1^. Minor variations in the intensity of the amide-II peak were also noticed when the spectra were normalized at 900 cm^−1^ for comparison (data not shown).

Next, we noticed both changes in the intensity of the amide-I peak as well as a shift toward either higher or lower frequencies after irradiation of BSA, BSF, or HSA prepared in either water or HEPES buffer (Fig. 4C–H, insets). From the FSD analysis of the amide-I peaks seen with the albumin solutions (Tables 5 and 6), we observed that the content of β-turn obtained for both control, non-irradiated BSA (18.4% for BSA in water and 16.9% for BSA in HEPES buffer) and HSA (16.6% for HSA in water and 9.6% for HSA in HEPES buffer) were comparable to the values reported earlier in the literature (13% for BSA as shown in ref. [81]; 7–14% for HSA in refs. [82], [83]). On the other hand, the content of α-helices and β-sheets for these two proteins differed considerably from the values reported previously by other groups. For instance, the content of α-helices was reported to be around 66% for BSA [81], [84], whereas we obtained lower values of either 12.8% for BSA prepared in water or 24.4% for BSA in HEPES buffer (Tables 5 or 6, respectively). Similarly, the content of β-sheets in BSA was reported to be near 20% [81], while we obtained a higher value of 48.8% for BSA prepared in water (Table 5). For BSA prepared in HEPES buffer, the content of β-sheets reached a value of 19.7% (Table 6), which was closer to the value of 20% reported in the literature [81]. For HSA, the content of α-helices reported earlier was shown to represent around 54 to 55% of the secondary structures of this protein, while β-sheets were reported to represent 17–30% [82], [83], which differed significantly from the values that we obtained for both HSA in water and HSA in HEPES buffer (Tables 5 and 6, respectively). As for BSF, we were unable to find studies describing its secondary structure.

**Table 5 pone-0010343-t005:** Secondary structures of serum protein solutions prepared in water before and after γ-irradiation as determined by FTIR analysis.

		Percentage of Secondary Structure of Serum Proteins after Irradiation Treatment		
		0 kGy	5 kGy	30 kGy
**BSA**	**α-Helix**	12.8	8.1	24.3
	**β-Sheet**	48.8	57.3	49.1
	**β-Turn**	18.4	19.5	17.1
	**Random Coil**	20.0	30.0	9.5
**BSF**	**α-Helix**	25.2	23.9	14.5
	**β-Sheet**	48.9	24.1	15.7
	**β-Turn**	13.6	2.9	7.6
	**Random Coil**	12.3	49.1	62.2
**HSA**	**α-Helix**	6.9	4.3	14.1
	**β-Sheet**	16.1	17.1	33.7
	**β-Turn**	16.6	18.7	24.8
	**Random Coil**	60.4	59.7	27.4

The deconvolution analysis of amide-I peaks was performed as described in the *Materials and Methods*.

**Table 6:** Secondary structures of serum protein solutions prepared in HEPES buffer before and after γ-irradiation as determined by FTIR analysis.

		Percentage of Secondary Structure of Serum Proteins after Irradiation Treatment		
		0 kGy	5 kGy	30 kGy
**BSA**	**α-Helix**	24.4	33.1	9.9
	**β-Sheet**	19.7	54.9	46.3
	**β-Turn**	16.9	4.5	16.8
	**Random Coil**	39.3	7.5	27.0
**BSF**	**α-Helix**	26.7	25.1	26.8
	**β-Sheet**	55.9	58.3	22.8
	**β-Turn**	3.1	8.6	1.8
	**Random Coil**	11.3	8.0	48.6
**HSA**	**α-Helix**	29.1	8.5	11.8
	**β-Sheet**	46.3	25.1	19.2
	**β-Turn**	9.6	30.2	16.7
	**Random Coil**	15.0	36.2	52.3

The deconvolution analysis of amide-I peaks was performed as described in the *Materials and Methods*.

As for the three proteins prepared either in water or in HEPES buffer and that had been γ-irradiated with either 5 kGy or 30 kGy, significant variations in secondary structure content could be inferred using the FSD analysis (Tables 5 and 6, respectively). However, these variations did not seem to follow any clear pattern that can be deemed interpretable at this time (Tables 5 and 6).

Taken together, our observations suggest that γ-irradiation largely altered the secondary structures of serum proteins. Nonetheless, we observed that the changes of secondary structures at the level of the individual proteins examined did not correlate with the doses of irradiation used. It is important to note that the lack of dose-dependent effects of γ-irradiation on protein secondary structures was also observed earlier under similar experimental conditions. For instance, Dogan *et al*. [79] reported that, while the random coils of hazelnut proteins gradually increased following γ-irradiation from 1.5 to 10 kGy, neither β-sheets nor β-turns followed any predictable dose-dependent variations in the irradiated proteins.

Moreover, treatments like lyophilization and thermal aggregation were shown to increase the degree of order for various proteins (e.g. bovine pancreatic trypsin inhibitor and bovine RNase A), as illustrated by an increase of β-sheets and a decrease of disordered, random coils [85]–[87]. Since our protein solutions were prepared from lyophilized proteins, it is possible that their origin as well as the buffers and aqueous solutions used to solubilize them may have accounted for the differences seen between our inferred data and those published to date for both BSA and HSA [81]–[84]. Moreover, deuterated water (D_2_O), which was not utilized here, is often used to remove interference produced by water during the analysis of the amide-I peak [77]. That is, while the strong O–H bending absorption peak of normal water overlaps with the amide-I band of proteins, there is no overlap when D_2_O is used since the O–H bending peak of D_2_O is found at a lower frequency [77]. In addition, the use of protein solutions of high concentrations (i.e. above 20 mg/ml) can provide spectra with higher signal-to-noise ratios, thereby reducing the effects of the background noise during FSD analysis [77].

While it appears that γ-irradiation may have produced extensive changes in the secondary structures of serum proteins, it is unclear why there was such high data scatter and dose-effect inconsistency (Tables 4–6). One obvious possibility may be due to extensive protein degradation occurring presumably as a result of the ionizing effects of γ-irradiation, an occurrence that could have introduced significant aberrations in the serum protein composition, not to mention secondary structure content distortion and unpredictability. This possibility was assessed next.

### Degradation of Serum Proteins Following γ-Irradiation as Shown by Sodium Dodecyl Sulfate-Polyacrylamide Gel Electrophoresis

**Figure 5:**
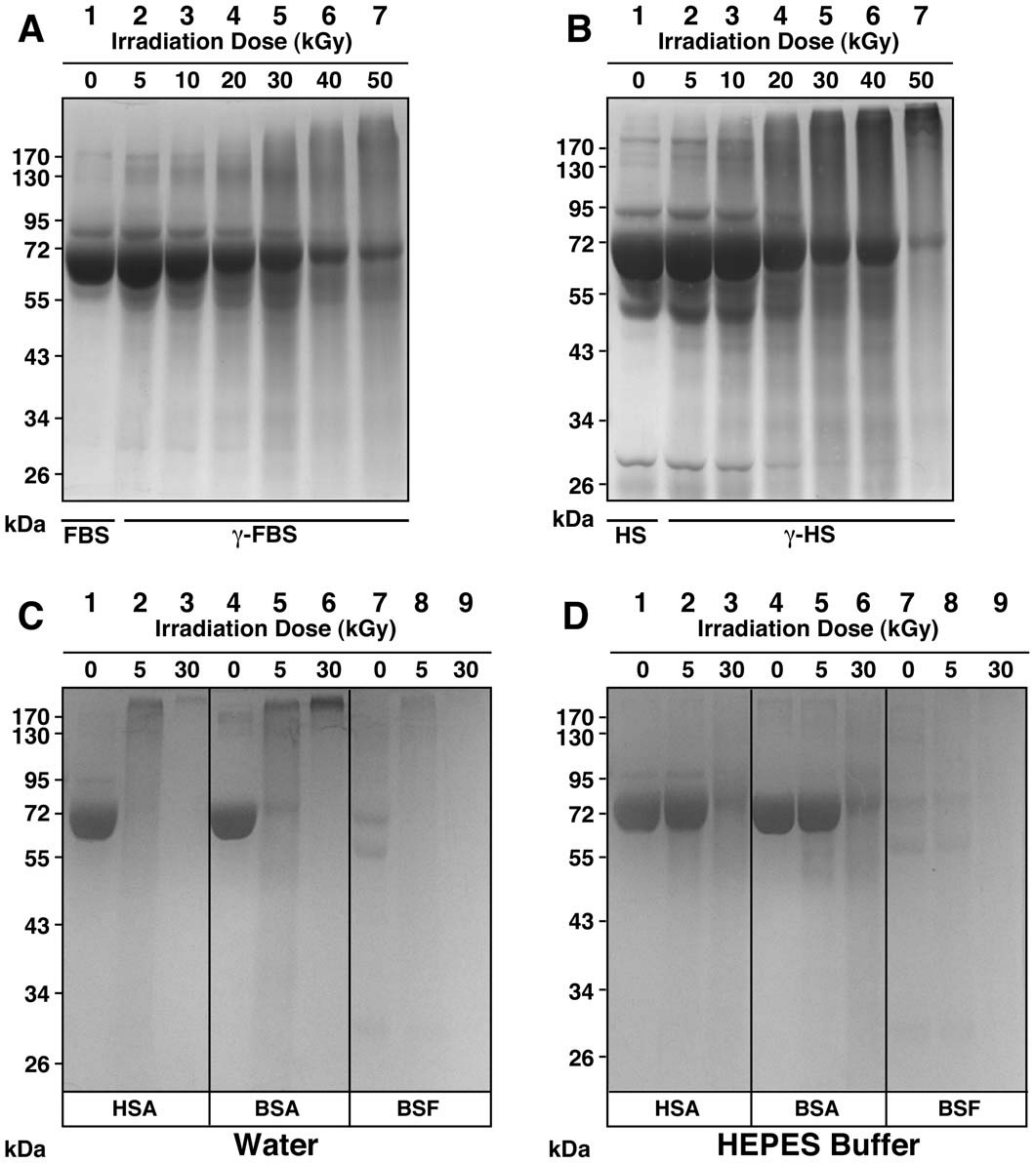
γ-Irradiation induces the degradation of serum proteins as revealed by SDS-PAGE. Equal volumes of (A) FBS and (B) HS, either untreated (“0 kGy”) or γ-irradiated at the doses indicated, were electrophoresed on a 10%-polyacrylamide gel in denaturing and reducing conditions. γ-Irradiation produced a dose-dependent degradation of serum proteins as seen through the gradual disappearance of the main protein bands as well as the progressive protein smearing observed in lanes 2 to 7 for both gels. Solutions of HSA, BSA, and BSF, prepared in either (C) water or (D) HEPES buffer were also electrophoresed either before or after γ-irradiation at either 5 or 30 kGy. Note the significant protein profile changes seen as a result of γ-irradiation and the protein solvent used.

In order to verify the extent of protein degradation produced by γ-irradiation, we examined the protein profile of the γ-irradiated sera by using sodium dodecyl sulfate-polyacrylamide gel electrophoresis (SDS-PAGE). A fixed volume of each serum solution was electrophoresed in denaturing and reducing conditions using a 10% polyacrylamide gel, followed by Coomassie blue staining (see *Materials and Methods*). The gel profile of non-irradiated FBS was shown to consist of a multitude of bands (data not shown) which, upon extensive dilution, revealed predominantly a strong band at 60–70 kDa and several other weaker bands at 170, 80, and 55–60 kDa, as well as a faint band of 28–32 kDa (Fig. 5A, lane 1). This protein profile was virtually identical to others obtained earlier for different lots of FBS processed under similar dilution conditions (see lane 5 of Fig. 12 of ref. [2]). In that previous study [2], we identified the major proteins bands observed in FBS by using in-gel trypsin digestion and tandem MALDI-TOF mass spectrometry (MS). The main proteins bands found in the profile of FBS included albumin (BSA, 60–70 kDa) and apolipoprotein A1 (28–32 kDa). Fetuin-A was not identified in the region of 55–60 kDa but it turned out to be one of the main protein species found in the scaffold of NB/NLP derived from FBS [2].

In the case of γ-FBS, there was a progressive smearing of bands with the major protein bands seen to gradually decrease in intensity with increasing doses of γ-irradiation (Fig. 5A, lanes 2 to 7). At 40 and 50 kGy, the main protein band of 60–70 kDa had virtually faded into the smeared background (Fig. 5A, lanes 6 and 7). Thus, while the main protein bands gradually disappeared into the background with increasing doses of γ-irradiation, the protein smears clearly increased in intensity, revealing both high and low molecular weight bands that were not seen in the control, non-irradiated FBS (Fig. 5A). While the low molecular weight bands may represent ionization-induced breakdown products, the high molecular weight bands, on the other hand, may have consisted of aggregated or crossed-linked proteins or fragments derived from γ-irradiation.

For HS, a somewhat similar protein profile was observed (Fig. 5B, lane 1) in line with earlier observations (see Fig. 12 of ref. [2]). The major protein bands of HS were identified earlier and were found to be equivalent to those found in FBS, with the human forms of albumin and apolipoprotein A1 being identified at molecular weights of 60–70 and 28–32 kDa, respectively, while fetuin-A was similarly identified as a 50–60 kDa protein species enriched in the scaffold of NB/NLP derived from HS [2].

The SDS-PAGE of γ-HS also revealed a progressive γ-dose-dependent decrease in the intensity of the major protein bands that was however accompanied by a progressive smearing of the gel lanes (Fig. 5B, lanes 2 to 7). Thus, similar to what had been observed with γ-FBS, while the major bands appeared to fade with an increase in the γ-irradiation doses used, the smeared background appeared to increase in intensity in the same gel lanes, suggesting in either a progressive breakdown or a cross-linking of protein fragments as a result of increasing doses of radiation (Fig. 5B, lanes 2 to 6). Band smearing appeared to reach a maximal intensity with 40 kGy (Fig. 5B, lane 6). With 50 kGy, the smear appeared to have faded drastically in intensity while a much more prominent high molecular band was noticed at the top of the gel lane, presumably consisting of aggregated or cross-linked proteins (Fig. 5B, lane 7).

These observations indicate that γ-irradiation induces considerable degradation of FBS and HS proteins that is not only dose-dependent but it also appears to occur in a non-specific manner. These degradations appear to be consistent with earlier findings of similar effects of γ-irradiation on both purified horse myoglobin [88] and hen protein mixtures found in eggs [89].

We next analyzed by SDS-PAGE the effects of γ-irradiation on the same purified protein solutions studied before. Both HSA and BSA dissolved in water produced a large band of 62–75 kDa (Fig. 5C, lanes 1 and 4, respectively). Following γ-irradiation of these proteins at 5 kGy, the prominent bands had faded considerably, being replaced instead by a smear covering a wide range of molecular weights (Fig. 5C, lanes 2 and 5). After γ-irradiation at a higher dose, i.e. 30 kGy, both the 62–75 protein bands and the background smears had largely disappeared, being replaced by a protein band of higher staining intensity at the top of each lane, indicating probably further aggregation or cross-linking of the remaining protein fragments (Fig. 5C, lanes 3 and 6).

In the case of BSF dissolved in water, the control, non-irradiated protein appeared to produce two bands at 55 kDa and 70 kDa (Fig. 5C, lane 7). Variation in the migration of fetuin-A and the presence of multiple bands in its gel profile were attributed earlier to the high level (30%) of glycosylation seen with this protein [90]. When the BSF was γ-irradiated at 5 kGy, both bands had largely disappeared while a faint protein smear was produced at the top of the lane (Fig. 5C, lane 8). However, with a γ-dose of 30 kGy, even this faint smear associated with 5 kGy had completely disappeared (Fig. 5C, lane 9).

For the proteins dissolved in HEPES buffer, we noticed considerable differences (Fig. 4D). For example, both HSA and BSA produced strong bands of 65–80 kDa (Fig. 5D, lanes 1 and 4). Following γ-irradiation at 5 kGy, the bands corresponding to both HSA and BSA remained essentially intact, while a faint protein smear could be distinguished below 65 kDa in both cases, indicating minimal or partial protein breakdown under these conditions (Fig. 5D, lanes 2 and 5). However, γ-irradiation of both HSA and BSA at 30 kGy decreased considerably the intensity of the main bands, producing in their place protein smears of broad molecular weight distribution, all of which appeared to reflect significant protein degradation (Fig. 5D, lanes 3 and 6).

When BSF was likewise prepared in HEPES buffer, we observed two bands of 55 kDa and 72 kDa (Fig. 5D, lane 7). γ-Irradiation of this BSF at 5 kGy produced a slight fading of these two bands (Fig. 5D, lane 8) while γ-irradiation at 30 kGy resulted in further fading of staining in this gel lane (Fig. 5D, lane 9).

Compared to the γ-irradiated proteins prepared in water, it was apparent that the HEPES buffer conferred partial protection against γ-irradiation. This conclusion is consistent with the protective role of HEPES buffer on serum proteins observed in previous sections through other types of measurements.

In summary, these results illustrate that, in addition to inducing conformational changes and protein unfolding (Fig. 3), along with pronounced changes of secondary structures (Fig. 4), γ-irradiation also produces considerable degradation of serum proteins (Fig. 5). Furthermore, this protein degradation is both γ-dose-dependent and non-specific, affecting apparently a broad array of proteins found in the serum. Specifically, at the sterilizing γ-dose of 30 kGy used by all the NB proponents to date, there is considerable breakdown of serum proteins that can be reproduced and confirmed with the purified proteins albumin and fetuin-A.

### γ-Irradiated Serum Retains its Ability to Seed NB and NB-like Particles in Culture

Given the fact that a long incubation time of several weeks to months in culture was required to demonstrate the presence of NB in body fluids or tissues and that only low amounts of particulate precipitates or aggregates were obtained even with such prolonged incubations, the early NB proponents sought to develop methods to enhance NB formation in culture [6]–[8]. γ-FBS that had been irradiated at 30 kGy was thus chosen with the assumption that this supplement would enhance NB formation [6]–[8], [30], [39], [40]. The use of γ-FBS as a feeder was founded on the observation that this solution apparently no longer produced NB after incubation under the conditions tested [7], [39]. In order to verify these assertions, we diluted each γ-irradiated serum at final concentrations ranging from 0.1 to 10% into Dulbecco's modified Eagle's medium (DMEM), and incubated these solutions in cell culture conditions for several months. After incubation, the presence of NB/NLP was monitored by both visual inspection and A_650_ turbidity readings as outlined elsewhere [6], [7], [39].

**Figure 6:**
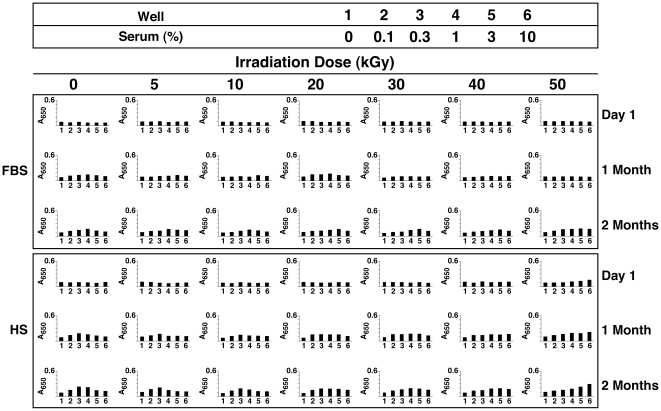
γ-Irradiated serum retains its ability to seed NB/NLP in culture. Aliquots of untreated FBS (“0 kGy”) and γ-FBS (“5-to-50 kGy”) were inoculated into DMEM to concentrations indicated on the top. A_650_ turbidity readings were performed following inoculation (“Day 1”) or after incubation in cell culture conditions for “1 Month” or “2 Months.” Control, untreated HS and γ-HS were treated in the same way (bottom panel). Notice the time-dependent turbidity increases seen for both non-irradiated and γ-irradiated sera that appear either as bell-shapes or straight lines.

As seen in Figure 6, with A_650_ readings shown as a function of the time of incubation, the inoculation of either non-irradiated FBS or the various γ-FBS samples into DMEM did not produce any immediate changes in optical density when compared to DMEM alone (Fig. 6, FBS panel, “Day 1”). With additional incubation and in the case of control, non-irradiated FBS, we have consistently seen a small increase in turbidity that could be detected by 1 and 2 months of incubation (Fig. 6, FBS panel, “0 kGy,” “1 and 2 Months”). Notably, the increase in turbidity was bell-shaped, with peak turbidity in this case achieved at 0.3% of FBS after 1 month of incubation and with a rightward shift of this same peak to 1% of FBS after 2 months. These results were identical to all previous findings made with serum inoculation [2]–[4], and the bell-shaped dose-dependent relationship had earlier been interpreted as supporting a dual inhibition-seeding mechanism for particle formation in which the inherent inhibitory influences within serum were seen to predominate initially, that is, with higher serum levels inducing in stronger inhibition of NB/NLP formation. Likewise, the slow rightward shift of the peak turbidities as a function of incubation time was interpreted as a gradual release or override of the same inhibitory mechanisms inherent in the serum, such that the precipitation and crystallization of calcium-and-apatite-containing NB/NLP are finally de-repressed [2]–[4]. Thus, with time, any serum inoculum will become increasingly prone to seeding NB/NLP. Remarkably, however, even with prolonged incubation, in this case up to 2 months, this “de-repression” is only partial and it is clear that inhibitory influences protecting the serum from overt calcification are still largely predominant even after this time, in line with findings made earlier [2], [4].

Unexpectedly, turbidity increases corresponding to particulate precipitation were also noticed for all the γ-FBS samples tested, irrespective of the radiation doses used, and these increases became invariably noticeable by 2 months of incubation (Fig. 6, FBS panel). For some inoculations, done with γ-FBS that had been obtained with lower radiation doses in the range of 5-to-20 kGy, the turbidity increases were clearly evident after 1 month of incubation (Fig. 6, FBS panel). That is, turbidity increases with γ-FBS that had been obtained with higher doses of radiation (30-to-50 kGy) took longer time to develop but were nonetheless visible by both visual inspection (not shown) and A_650_ reading (Fig. 6, FBS panel) after 2 months. The turbidity increases seen for the γ-FBS inoculations were also shown to follow bell-shape curves similar to those of control, non-irradiated FBS. In all the samples studied, there was also a slow rightward shift of the peak turbidities with increased time of incubation (i.e. 2 months vs. 1 month). For example, with γ-FBS that had been irradiated with 20 kGy, turbidity was seen to peak at 1% γ-FBS after 1 month of incubation, shifting thereafter to peak at 3% γ-FBS by 2 months (Fig. 6, FBS panel, “20 kGy”). As pointed out earlier, these observations are compatible with a dual inhibition-seeding activity inherent to the serum [2], [4]. Surprisingly, such bell-shape curves of precipitation were noticed with γ-FBS irradiated even at the higher doses of 30 to 50 kGy, all of which gave peak turbidities with 3% γ-FBS (Fig. 6, FBS panel, “2 Months”). Together, these results indicate that in spite of γ-irradiation, FBS had retained largely the ability to seed NB/NLP, contrary to what would have been expected from the vast literature that had used γ-FBS as a sterilized feeder for NB cultures and with itself deemed to be completely devoid of any NB-seeding potential (see Table 1 for a list of such papers). While the higher doses of γ-irradiation used (30-to-50 kGy) appeared to suppress somewhat NB/NLP formation produced by the resultant γ-irradiated FBS, these same γ-irradiated sera were nonetheless fully capable of seeding NB/NLP with longer incubations (Fig. 6, FBS panel). Moreover, the dose-dependent, bell-shaped turbidity increases seen with all γ-FBS inocula studied, irrespective of the dose of γ-irradiation used (5-to-50 kGy), reveal that these same irradiated sera have largely retained their NB/NLP inhibitory and seeding potential.

Similar findings were obtained with both HS and γ-HS used as the seeding inocula (Fig. 6, HS panel). However, there were a few minor differences. For example, the γ-HS that had been obtained with 50 kGy produced immediate low turbidity changes when added to DMEM to a final concentration of 1-to-10% (Fig. 6, HS panel, “50 kGy,” “Day 1”). This result was seen to be due to the presence of precipitates in the γ-HS that had been irradiated at 50 kGy, an observation which was also apparent by visual inspection, as noted earlier (Fig. 1A, HS row, “50 kGy”). Regardless, all γ-HS samples studied were able to produce turbidity increases after 1 month of incubation that were comparable to those seen with control, non-irradiated HS (Fig. 6, HS panel, “1 Month”). In this case, precipitation was noticed even with the γ-HS samples irradiated at doses of 30 to 50 kGy. Turbidity continued to increase with the length of incubation (2 months). Clearly, this slow increase of turbidity cannot be explained by the sole presence of particulate matter seen in γ-HS that had been irradiated at 30 to 50 kGy, which accounted only for the initial low turbidity changes. Notably also, there was a less noticeable rightward shift in peak turbidity with prolonged incubation here as compared with γ-FBS (Fig. 6, compare “1 Month” vs. “2 Months” seen for both FBS and HS). In fact, with γ-HS, this rightward shift in peak turbidity was only apparent with the higher doses of γ-irradiation used (40 and 50 kGy).

These results indicate that both γ-FBS and γ-HS can lead to the slow formation of NB-like precipitates, contradicting directly earlier observations [6]–[8], [39]. Given the slow, bell-shaped, dose-dependent precipitation produced by serum—be it control or γ-irradiated—when inoculated into culture medium, it is possible that the earlier studies using γ-FBS irradiated with 30 kGy [6]–[8], [39] had somehow highlighted only the inhibitory influences of γ-FBS, a possibility supported by the fact that relatively large amounts of serum (10%) were used in those studies. Nonetheless, a more careful evaluation of γ-irradiated serum, as exemplified here, would certainly have revealed the glaring contradictions and fallacies involved.

### Inhibition of NLP Formation by γ-Irradiated Serum

We had shown earlier that this slow formation of NB/NLP in culture medium as a result of serum inoculation, generally taking several weeks to reach detectable precipitation, was the source of a large data scatter which may have resulted in the kind of spurious data associated with the controversial NB biology [2]. We further demonstrated that this same precipitation reaction could be speeded up considerably with the addition to medium of supersaturating concentrations of calcium and phosphate, a reaction that resulted in marked precipitation within hours or at most a few days [2]. We also noticed that both FBS and HS inhibited the formation of NLP in medium inoculated with these same supersaturating concentrations of calcium and phosphate [2]. That is, while both FBS and HS induced by themselves the slow formation of NB/NLP when added to DMEM, as also exemplified by the experiments shown in Figure 6, both sera inhibited the much faster formation of NLP produced in DMEM by the exogenous addition of the precipitating ions calcium and phosphate to supersaturating levels. We observed further that this same inhibitory effect of serum was sensitive to the protease trypsin, implicating a role for serum proteins in this inhibitory process [2]. It was also apparent that the inhibitory effect of serum on NLP was only transient and that it could be gradually and spontaneously overcome with time [2]. This “precipitation-inhibition” assay thus allowed us to study the dual inhibition-seeding effect associated with serum.

**Figure 7:**
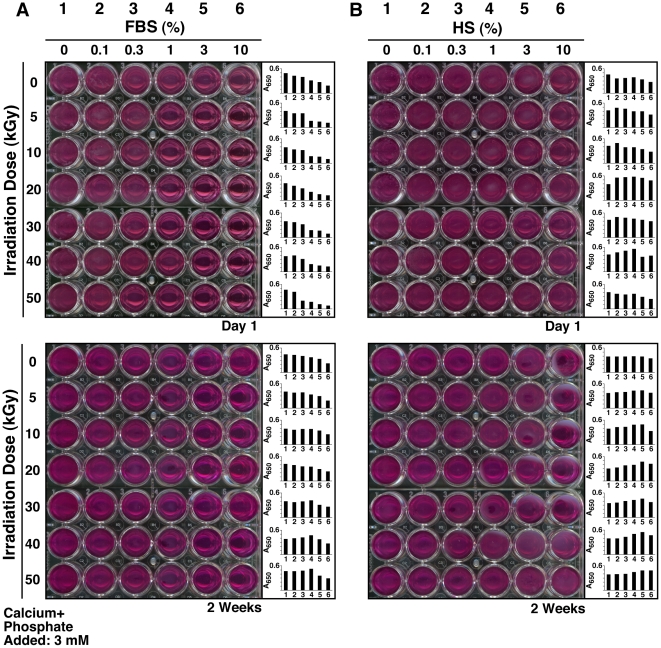
Transient inhibition of NLP formation by γ-irradiated serum. (A) NLP were prepared from 3 mM each of CaCl_2_ and NaH_2_PO_4_ in DMEM containing either untreated FBS or γ-FBS at the final concentrations indicated on the top. A_650_ turbidity readings were performed following inoculation (“Day 1”) or after “2 weeks” of incubation in cell culture conditions. (B) Similar experiments were performed with untreated/γ-irradiated HS. Both the untreated sera and the γ-irradiated sera were shown to inhibit NLP formation in a dose-dependent manner (“Day 1”). By 2 weeks, this same inhibitory effect of serum was partially overcome.

In order to verify whether γ-irradiation influenced this same inhibitory effect of serum, we performed precipitation-inhibition assays whereby the γ-irradiated sera were diluted at various concentrations into DMEM, followed by the addition of 3 mM each of the precipitating ions calcium and phosphate [2]. The use of this supersaturating amount of precipitating ions (3 mM) allowed for the immediate observation of significant precipitations, thereby making it easier to quantify for any inhibitory influences being studied. Concordant with our previous results [2], untreated FBS was shown to readily inhibit NLP formation in a dose-dependent manner (Fig. 7A, “0 kGy;” note the decrease of turbidity with increasing serum concentrations). Surprisingly, the γ-FBS solutions irradiated at various doses were also shown to inhibit NLP formation in the same manner (Fig. 7A, “5-to-50 kGy”). Moreover, when we compared the inhibitory activities of the various serum solutions used in these experiments, we noticed that the various γ-FBS solutions actually inhibited precipitation more strongly than the untreted FBS under similar conditions (Fig. 7A, “5-to-50 kGy” vs. “0 kGy”).

As demonstrated by the experiments shown in Figure 6 and still in line with earlier results [2], the inhibition of NLP formation induced by FBS was only transient and could be partially overcome with time as seen after incubation in cell culture conditions for 2 weeks (Fig. 7A). Notably, the inhibitory effect of γ-FBS was also partially released after this period of incubation (Fig. 7A). Taken together, these observations indicate that, in spite of γ-irradiation having induced extensive damage on serum proteins, it surprisingly did not alter the dual inhibition-seeding characteristics of FBS.

The inhibitory activity of γ-HS was also studied using the same precipitation-inhibition assays (Fig. 7B). As noted earlier [2], the inhibitory activity of control, non-irradiated HS was found to be lower than that of FBS (Fig. 7, compare the two “0 kGy” horizontal lanes, HS vs. FBS panels). This phenomenon had been attributed at least in part to FBS containing a much higher concentration of fetuin-A, considered a potent inhibitor of calcium phosphate mineralization [2]–[4], [91]–[94]. Unlike γ-FBS, which inhibited this type of NLP formation more strongly than control FBS, both γ-HS and untreated HS produced comparable levels of NLP inhibition (Fig. 7B). These results indicate that the inhibitory mechanisms inherent in HS, directed against the formation of NLP in the presence of supersaturating levels of calcium and phosphate, remain largely unchanged following the γ-irradiation of HS.

Like the results seen with FBS, the inhibitory effect of HS on NLP formation was also transient, being eventually overcome with time as seen after incubation for 2 weeks (Fig. 7B). Similarly, the inhibitory effect of γ-HS was also released after this incubation period (Fig. 7B, “2 Weeks”). In this case, concentrations of γ-HS at 1 to 10% appeared to seed more particles than lower concentrations after 2 weeks (Fig. 7B, “5-to-50 kGy,” “Day 1” vs “2 Weeks”). This general seeding pattern seen with γ-HS as a function of the size of the serum inoculum had earlier been reported for untreated HS incubated under similar conditions [2]. These observations demonstrate that the inhibitory-seeding activities of both FBS and HS remain largely intact following γ-irradiation at the doses studied.

Given the extensive serum protein breakdown produced by γ-irradiation as evidenced by SDS-PAGE (Fig. 5A and B), these results were initially deemed surprising since serum proteins had been shown earlier to be required in order for the inhibition-precipitation assays to work [2]. Our results are all the more intriguing since γ-irradiation is shown here to induce extensive protein degradation in a dose-dependent manner and, yet, at the maximal irradiation dose of 50 kGy used, shown to produce significant breakdown and smearing of proteins as evidenced by SDS-PAGE (Fig. 5), the inhibitory activities for both γ-FBS and γ-HS appear to have become enhanced compared to both control sera as well as γ-irradiated sera obtained with lower irradiation doses (Fig. 7). In fact, for γ-FBS, its inhibitory activity appears to increase paradoxically with increasing doses of γ-irradiation (Fig. 7A). Presumably, the serum protein fragments induced by the γ-irradiation treatments may have in fact retained their ability to bind to calcium phosphate crystals. It is possible, and perhaps even likely, that the fragments of the calcium and apatite binding proteins, due to their smaller size or surface exposure, may now interact more efficiently with the growing crystals compared to the larger native proteins present in control, non-irradiated serum! In turn, this enhanced access to binding by the nascent apatite structures may presumably result in an equally effective inhibition or abortion of further crystallization. This concept relies and builds on the well-established notion that the binding of proteins or other macromolecules onto nascent apatite crystals usually prevents further calcium phosphate deposition and leads to inhibition of crystal growth [95], [96]. However, while our main focus here has dwelled on proteins and their potential fragments, it is entirely possible that other degraded serum components, like lipids or polysaccharide molecules, may also have contributed to this inhibitory process.

Our results show further that, at best, these same inhibitory effects are only transient and that the inhibition of NB/NLP formation induced by both γ-irradiated FBS and HS is released or de-repressed over time and in a manner largely similar to that seen with control, untreated serum. Thus, both the inhibitory and seeding properties of serum appear to have been left largely intact following γ-irradiation, despite the extensive breakdown of serum proteins seen associated with the irradiation treatment.

### Protein Profiles of NLP Prepared from γ-Irradiated Serum

To verify whether the fragmented proteins produced by γ-irradiation could still bind to NLP, we examined the protein profiles of the particles prepared in the presence of 10% γ-irradiated serum as described in the previous section (Fig. 7). The particles were prepared by adding 3 mM of calcium and phosphate ions, followed by centrifugation to retrieve the particles and two washing steps with DMEM to remove unbound proteins. Next, the serum proteins that remained bound to NLP were recovered by dissolving the particles in 50 mM EDTA prior to processing for SDS-PAGE (see *Materials and Methods*).

**Figure 8:**
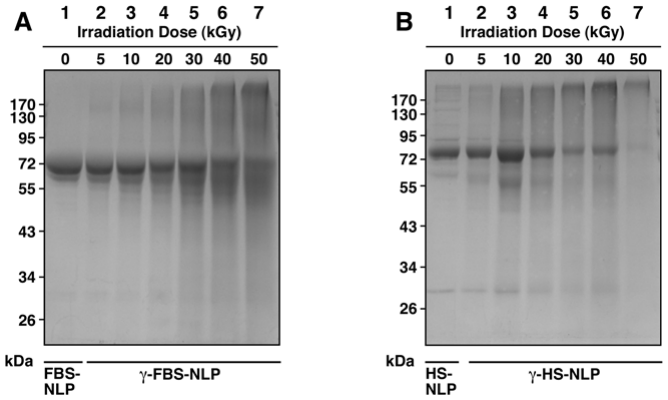
Protein profiles of NLP prepared from γ-irradiated serum. (A) NLP were prepared by adding 3 mM of both CaCl_2_ and NaH_2_PO_4_ into DMEM containing 10% of either untreated FBS (“FBS-NLP”) or γ-FBS (“γ-FBS-NLP”). (B) Particles were prepared in the same manner using either non-irradiated HS (“HS-NLP”) or γ-HS (“γ-HS-NLP”). After incubation, centrifugation and washing, the particles were resuspended into 50 mM EDTA, and processed for SDS-PAGE as described in the *Materials and Methods*. Note the progressively diffuse protein smears found in the NLP derived from serum that had been irradiated with increasing doses of γ-radiation.

With this procedure, we observed that the protein profile of NLP prepared in the presence of control, non-irradiated FBS (FBS-NLP) consisted of three distinct bands: a prominent band at 68–72 kDa, a minor band at 62–66 kDa and a faint band at 28–32 kDa (Fig. 8A, lane 1), a protein profile similar to that reported earlier [2]. There, the three bands were demonstrated to be albumin, fetuin-A, and apolipoprotein A1, respectively [2]. It should be noted however that in our earlier studies [2], [3] other minor bands could also be found in the scaffold of FBS-NLP that included bands at 95, 130, 170, and 190 kDa [2], [3]—a difference that can be largely attributed to the amount of proteins used to assemble the NLP, with a higher protein input producing not only stronger but also more bands. This assertion is confirmed by our earlier demonstration that the protein composition of FBS-NLP consisted largely of a mirror representation of the serum protein composition itself, with the main serum proteins also comprising the main protein constituents of the FBS-NLP scaffold [2], [3].

When γ-FBS was used instead to prepare for NLP, the protein profiles of the resulting particles (γ-FBS-NLP) showed a progressive fading of the main protein bands along with a smearing that increased with the γ-dose used (Fig. 5A, lanes 2–7, compare with lane 1). Remarkably, the protein profiles of these γ-FBS-NLP resembled largely the protein profiles of the whole γ-FBS shown earlier (Fig. 5A, lanes 2–7). Specifically, both diffuse low and high molecular weight bands were present in the γ-FBS-NLP that closely resembled the smeared bands seen with whole γ-FBS. It is clear from these data that both small serum protein fragments as well as large and presumably cross-linked aggregates present in γ–FBS bind avidly to the nascent mineral phase of the growing NLP such that they become concentrated in the scaffold of the resultant γ-FBS-NLP. This mechanism of protein adsorption by the nascent particles appears to be similar, if not identical, to the earlier mechanism of assembly associated with normal serum and serum proteins [2]–[4]. In both cases, calcium and apatite binding proteins from the serum—untreated or γ-irradiated—are seen to concentrate within the scaffold of the particles formed, in proportion to their serum concentrations as well as their calcium and apatite binding affinities [2], [3].

We observed similar results for NLP prepared from HS and γ-HS (Fig. 8B). That is, the scaffold of HS-NLP was found also to consist of a few major bands, the most prominent band being again the albumin band of 68–75 kDa, accompanied by several minor bands, including those of fetuin-A (52–65 kDa) and apolipoprotein A1 (27–33 kDa; see Fig. 8A, lane 1, and also refs. [2], [3] for other minor protein bands along with their protein identification). Like in the case of FBS-NLP, this composition also appeared largely as a reflection of the normal distribution of proteins found in the HS as well as a representation of their respective calcium and apatite binding affinities [2]. As for γ-HS-NLP, a progressive fading and smearing of the protein bands was also seen that was dependent of the γ-dose used (Fig. 8B, lanes 2–7). A protein smear of broad molecular weight distribution could also be seen with γ-HS-NLP, indicating a similar binding to NLP by protein fragments as well as aggregated or cross-linked proteins.

Taken together, our results indicate that, for both FBS and HS, γ-irradiation results in significant breakdown of serum proteins, and, yet, this breakdown leaves largely intact the ability of the protein fragments or aggregates to bind to nascent crystals prepared from the addition of supersaturating amounts of calcium and phosphate ions. Our results indicate that the increased inhibition activity of γ-irradiated serum may in fact be linked to the presence of these small protein fragments. That is, these protein fragments appear to participate in the same dual inhibition-seeding mechanisms underlying the assembly of NLP that had been described earlier for intact serum proteins [2]–[4]. Accordingly, these same fragments appear to be concentrated in the scaffold of NLP in a stoichiometric fashion, in direct proportion to their concentration in the serum milieu as well as their respective affinities shown for calcium and apatite.

### Seeding of NLP from γ-Irradiated Serum Proteins Inoculated in Metastable Medium Versus Medium Containing Submillimolar Amounts of Calcium and Phosphate Ions

In a previous study [4], we observed that the two serum proteins albumin and fetuin-A failed to induce mineralization when added either individually or in combination to metastable culture medium (i.e. DMEM). On the other hand, we found that the two proteins did enhance the formation of NLP in DMEM inoculated with supersaturing amounts of calcium and phosphate [4]. In fact, the formation of NLP was clearly enhanced in the presence of either one of these two proteins even when the DMEM was inoculated with submillimolar amounts of calcium and phosphate ions (i.e. at levels that did not result in precipitation on their own, without the addition of proteins) [4]. In order to verify whether γ-irradiation could affect this complex seeding process, we monitored the ability of γ-irradiated BSF and γ-irradiated BSA to form NLP in either metastable or supersaturated DMEM. It should be noted however that DMEM supersaturated with calcium and phosphate ions tend to precipitate on their own even without the addition of exogenous proteins, thereby making the role of proteins more difficult to quantify. We thus chose to inoculate DMEM only with submillimolar amounts of precipitating ions, i.e. at levels that either caused no precipitation or only borderline precipitation. For these same experiments, we used the protein solutions prepared in HEPES buffer since these solutions remained free of particulate matter following γ-irradiation (Fig. 1C).

**Figure 9:**
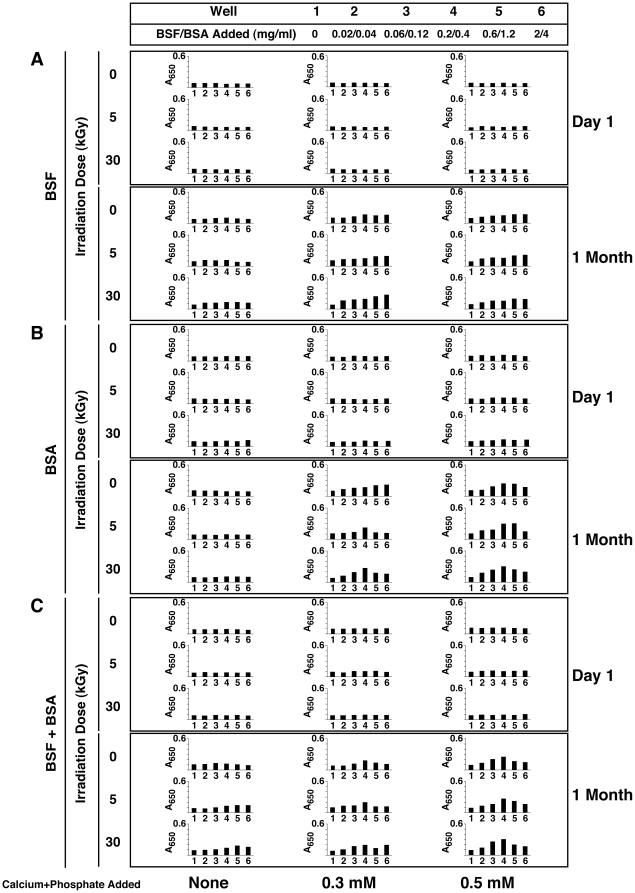
Formation of NLP from γ-irradiated serum proteins in metastable medium versus medium containing submillimolar amounts of calcium and phosphate. Solutions of (A) BSF, (B) BSA, or (C) both proteins, either untreated or γ-irradiated at the doses indicated on the left, were diluted into DMEM to the concentrations shown on the top. While “None” refers to the absence of additional ion input added to the metastable DMEM, the precipitating reagents CaCl_2_ and NaH_2_PO_4_ were added at either 0.3 or 0.5 mM in some experiments as indicated at the bottom (labeled as “Calcium+Phosphate Added”). A_650_ turbidity readings were monitored after incubation in cell culture conditions for either one hour (“Day 1”) or “1 Month” as indicated on the right.

We first inoculated the non-irradiated proteins into DMEM without additional ion input and incubated the solutions in cell culture conditions for at least 1 month (Fig. 9, “None” column, “0 kGy” rows). We used a wide range of protein concentrations, with BSF varying between 0.02 and 2 mg/ml and BSA between 0.04 and 4 mg/ml, attending to similar experiments described elsewhere [4]. There, the use of vastly different concentrations of BSF vs. BSA took into account the much higher apatite-binding and NLP-inhibitory activity associated with fetuin-A compared to albumin [4]. In accord with our earlier study, no turbidity increase was noted with either control BSF or control BSA, or a combination of both, inoculated into DMEM and incubated for 1 month. These negative results could not be attributed to the particular protein concentrations chosen for our experiments here since even wider ranges of concentrations used did not yield any turbidity increase after extensive incubations up to 2 months (data not shown; see also ref. [4] for experiments in which much higher levels of both BSF and BSA were used, all of which gave essentially the same negative results). These results were in line with the conclusion reached earlier [4] that neither BSA nor BSF, nor a combination of both, could seed directly mineral nanoparticles in metastable DMEM, even after prolonged incubation.

Of the two γ-irradiated proteins tested, only γ-BSF inoculated into DMEM produced low turbidity increases after 1 month of incubation, while γ-BSA induced no changes (Fig. 9A and B, “None” column, “5 and 30 kGy” rows). Consistent with this observation, a mixture of both γ-irradiated BSF and γ-irradiated BSA produced slightly higher turbidity increases after incubation for 1 month (Fig. 9C, “None” column, “5 and 30 kGy” rows). In this case, the turbidity increase was not only enhanced compared to the addition of γ-BSF alone but it was also shifted to the right, indicating additive effects seen in the presence of the two proteins. Moreover, the turbidity increase produced by γ-BSF appeared to be dependent on the dose of γ-irradiation used, with 30 kGy giving slightly higher precipitation as compared with 5 kGy (Fig. 9A and C, “None” column, “1 Month” row). Although the amount of NLP precipitation was relatively low under these conditions, the turbidity was seen to continue to increase steadily with time (data not shown).

Taken together, these results indicate that a significant conformational change may have been induced by γ-irradiation that now propitiates the formation of NLP by γ-BSF even under metastable conditions. On the other hand, γ-irradiation does not appear to be sufficient in inducing irradiated BSA to initiate NLP formation under these same conditions. These results are reminiscent of our earlier findings in which denaturation by heat treatment was sufficient to trigger BSA to initiate mineral aggregation and precipitation, but not BSF under similar heat-treatment conditions [4]. There, we had suggested the need for protein unfolding to ensue in order for the apatite binding sites to be more readily exposed, a transformative process which in our view might explain the dual NB/NLP inhibition-seeding mechanisms seen associated with serum proteins and other organic compounds [4]. However, we were unable to explain there why only BSA was heat-susceptible, driving it to seed NLP following heat treatment, while BSF remained largely heat-insensitive (i.e. it did not show any tendency to seed NLP after heat treatment) [4]. In the case of γ-irradiation studied here, on the other hand, γ-BSF, but not γ-BSA, was seen instead to induce NLP in metastable medium, suggesting in significant differences between these two proteins in response to various denaturing treatments.

We studied next the formation of NLP in DMEM solutions whereby small submillimolar amounts of calcium and phosphate ions were added. The addition of either 0.3 or 0.5 mM calcium and phosphate ions into DMEM, with or without proteins, did not result in immediate precipitation (Fig. 9, “Day 1”). Incubation of DMEM for up to 1 month in absence of proteins but in the presence of these added submillimolar amounts of calcium and phosphate ions did not yield in precipitation either (Fig. 9, wells 1, “0.3 mM and 0.5 mM” columns, “0 kGy and 1 Month” row). However, with the presence of submillimolar amounts (0.3 mM or 0.5 mM) of precipitating ions in DMEM, the subsequent inoculation of either untreated BSF or untreated BSA, or a combination of both, conferred a slow but progressive turbidity increase with incubation (Fig. 9, wells 2–6, “0.3 mM and 0.5 mM columns, “0 kGy and 1 Month” row). The turbidity increase in these solutions was seen to follow either a straight-line or a bell-shaped relationship with respect to the amount of protein added, and this variable response did not appear to correspond to either the particular protein species tested or the calcium phosphate ion concentrations used. This seemingly random variation was seen earlier with the other concentration ranges of proteins and precipitating ions studied [4]. Regardless, these results appear to confirm the conclusion reached earlier for both fetuin-A and albumin in that they appear to synergize with added calcium and phosphate ions to enhance the formation of NLP, suggesting a seeding effect produced by these proteins that is somehow revealed only in the presence of added precipitating ions [4].

The same DMEM containing either 0.3 mM or 0.5 mM of added precipitating ions was next inoculated with γ-BSF and/or γ-BSA. Proteins were irradiated with either 5 or 30 kGy. γ-BSF, γ-BSA, or a combination of both, produced turbidity increases that were largely similar to those produced by the control, untreated proteins under similar conditions (Fig. 9, “0.3 mM and 0.5 mM” columns, “1 Month” rows). We did not see any definitive pattern that could be used to distinguish between the results obtained with the different γ-irradiation doses (5 kGy vs. 30 kGy) or ion concentrations (0.3 mM vs. 0.5 mM of calcium and phosphate) used. Furthermore, as a response to inoculation with varying doses of γ-irradiated proteins, we observed both straight-line and bell-shaped dose-dependent increases in turbidity (Fig. 9). It is thus apparent from these experiments that γ-irradiation does not affect the ability of either BSF or BSA to “seed” NLP in the presence of supersaturating amounts of precipitating ions. It is also clear that γ-irradiation does not seem to change the “inhibitory” activity seen with high concentrations of these same proteins.

These observations would suggest further that γ-irradiation may have induced significant conformational changes in the structures of both fetuin-A and albumin as to allow these two proteins (or their fragments) to induce the seeding of NLP. This seeding propensity may be deemed analogous to that produced by the two untreated proteins seen in the presence of exogenously added calcium and phosphate ions, as depicted here as well as elsewhere [4]. Presumably, these two proteins may have become unfolded, or, more likely, fragmented, thereby exposing more effectively both calcium and apatite binding sites that are in turn involved in both inhibiting and seeding the formation of NLP. That is, the protein fragments in question are seen to act first as inhibitors of crystallization until they are overwhelmed or de-repressed by the continued presence and accumulation of precipitating ions until they become seeds or nidi for further apatite crystal growth, a transformative process consistent with the dual inhibitory-seeding mechanisms proposed by us for the assembly of NLP [2]–[4].

### Transient Inhibition of NLP Formation by γ-Irradiated Proteins in the Presence of Supersaturating Concentrations of Calcium and Phosphate Ions: Demonstration of a Dual Inhibition-Seeding Mechanism

**Figure 10:**
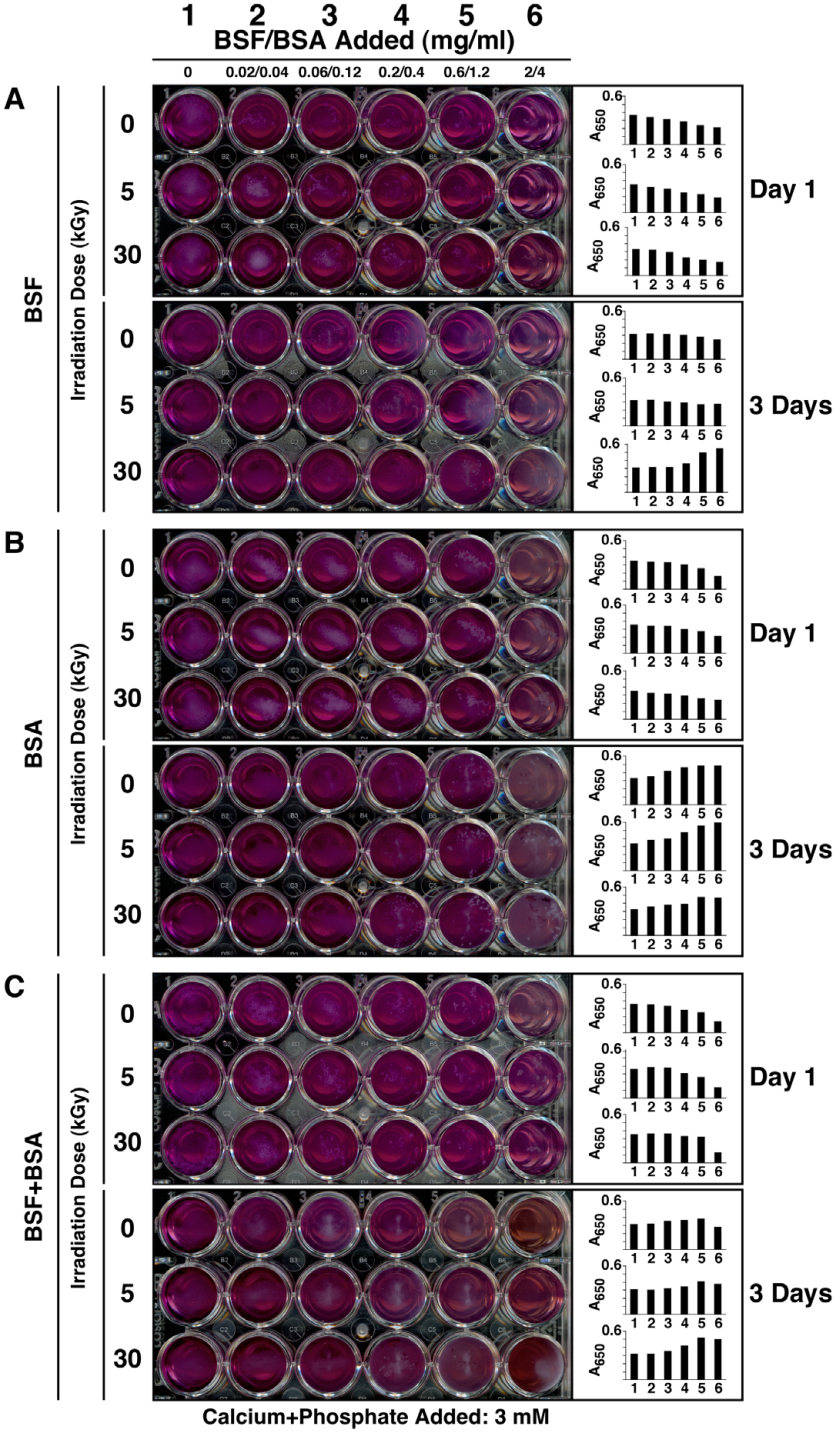
Transient inhibition of NLP formation by γ-irradiated serum proteins in supersaturated ionic medium. (A–C) NLP were prepared from 3 mM each of CaCl_2_ and NaH_2_PO_4_ added into DMEM containing either untreated or γ-irradiated forms of BSF and/or BSA at the final concentrations indicated on the top. The doses of γ-irradiation used are indicated on the left. The 24-well plates were photographed and A_650_ turbidity readings were performed after incubation in cell culture conditions for 1 hour (“Day 1”) or “3 Days” as indicated. Both non-irradiated (“0 kGy”) and γ-irradiated (“5 and 30 kGy”) proteins inhibited NLP formation in a similar, dose-dependent manner, but this inhibition appeared to have been partially overcome to varying degrees by 3 days of incubation. Note the marked differences in inhibition reversal between (A) BSF and (B) BSA. (C) The results seen with a combination of BSF and BSA appear as summations of the individual effects seen with these proteins.

The results shown here clearly indicate an enhanced seeding potential for both γ-fetuin-A and γ-albumin. However, it is unclear whether these γ-irradiated proteins can still “inhibit” effectively NLP formation produced by supersaturating amounts of calcium and phosphate ions. In the experiments done in the presence of submillimolar amounts of precipitating ions and shown in Figure 9, we had obtained bell-shaped turbidity increases suggestive of an inhibitory tendency at high protein concentrations, but a high margin of error was seen with different experiments and, in fact, some of the experiments shown for γ-BSF in Figure 9 displayed a straight-line increase of turbidity as a function of the protein amounts used. Apparently, the low amounts of precipitating ions added to DMEM may have accounted for this experimental variability. To address this issue more carefully, that is, in order to further verify whether γ-irradiation influences the inhibitory effect of either fetuin-A or albumin seen on NLP formation, we tested γ-irradiated proteins in the context of the same precipitation-inhibition assays shown in Figure 7. That is, we chose to add 3 mM of precipitating ions to DMEM in order to obtain immediate measurable precipitation that could in turn be tested against any potential inhibitory factors like γ-irradiated proteins. The γ-irradiated proteins were thus diluted into DMEM to the final concentrations shown in Figure 10, followed by the addition of 3 mM calcium and phosphate each (Fig. 10).

The addition of 3 mM calcium and phosphate ions to DMEM was seen to induce immediate precipitation (Fig. 10, wells 1). Under these supersaturating conditions, we were able to demonstrate next that either control BSF or control BSA, or a combination of both, inhibited NLP formation in a dose-dependent manner (Fig. 10, wells 2–6, “0 kGy”), similar to earlier findings [2], [4]. It should be pointed out that the BSF concentrations used in each well were some 6 times lower than the corresponding BSA concentrations, and yet the two proteins gave comparable inhibitions on the turbidity increase produced by the addition of 3 mM each of calcium and phosphate ions. These observations confirm again the well-established notion that fetuin-A is a much stronger inhibitor of calcification than albumin [2]–[4], [92]–[94].

We next tested the effects of γ-BSF and γ-BSA in the context of these same precipitation-inhibition assays. Both γ-irradiated proteins, by themselves and in combination, gave inhibition patterns largely comparable to those seen with the control, non-irradiated proteins (Fig. 10A–C, “Day 1”). In fact, γ-BSF produced slightly stronger inhibitions when compared with control BSF, whereas γ-BSA and BSA produced largely similar inhibitory profiles. When added together, however, the two γ-irradiated proteins did not produce any significant changes compared to their control counterparts. Moreover, as seen also with the control proteins, the inhibition produced by both γ-irradiated proteins increased as a function of the amounts of γ-irradiated proteins used. However, the results did not seem to vary significantly with the dose of γ-radiation used (i.e. 5 kGy vs. 30 kGy, Fig. 10, “Day 1”).

The dose-dependent inhibition of NLP formation produced by control BSA under supersaturating ion conditions appeared to have been overcome after incubation in cell culture conditions for 3 days (Fig. 10B, “3 Days”). With 3 days of incubation, BSA appeared to induce a dose-dependent increase in precipitation that corresponded linearly to the amount of BSA present in medium. This inhibition reversal stood in marked contrast to that seen with BSF by day 3, which continued to show significant inhibitory influences even at the high protein concentrations used (Fig. 10A, “3 Days”). As for the combination of BSF and BSA, this inhibition reversal appeared to be partial, as if it represented a summation of individual effects produced by these two proteins, with the resultant bell-shaped dose-dependent relationship indicating an override of calcification inhibition at low protein levels while these same inhibitory influences are retained with the higher protein concentrations (Fig. 10C, “3 Days”). These results are in line with earlier data that also showed BSF as a much more potent calcification inhibitor than BSA when tested in similar precipitation-inhibition assays [4]. Remarkably, the inhibition produced by relatively low amounts of BSF under supersaturating ion conditions could be seen to have been sustained for at least 1 month of incubation [4].

The inhibitory effects exerted on NLP formation by γ-BSA that had been irradiated with either 5 kGy or 30 kGy were also partially released after 3 days of incubation (Fig. 10B). In marked contrast, γ-BSF showed a much more complex response that appeared to vary with the dose of γ-irradiation used (Fig. 10A, “3 Days”). Thus, when irradiated at 5 kGy, γ-BSF showed partial inhibition reversal, while the 30 kGy-γ-BSF clearly showed a reversal that appeared practically complete (Fig. 10A, “3 Days”). In fact, with 30-kGy γ-BSF, the initial dose-dependent increase in inhibition seen with both control BSF and 5-kGy γ-BSF can now be characterized by a steep dose-dependent increase in seeding. As expected, with a combination of γ-BSF and γ-BSA, an intermediate level of inhibition reversal could be seen that appeared to represent a summation of effects produced by both proteins individually (Fig. 10C, “3 Days”).

These results reveal important differences between albumin and fetuin-A insofar as their calcification inhibition-seeding influences are concerned that appear to be further accentuated by γ-irradiation. It is apparent that fetuin-A is a potent inhibitor of calcification under supersaturating conditions and that this inhibition is only released with high doses of γ-irradiation (30 kGy), whereas albumin tends to exert stronger seeding influences with or without γ-irradiation. Our results further demonstrate that the dual inhibition-seeding characteristics, seen with both control and γ-irradiated serum (Fig. 7), can be replicated fully at the level of untreated and γ-irradiated serum proteins albumin and fetuin-A. At the level of these two serum proteins tested, it is clear that this dual inhibition-seeding characteristic is altered (or released) partially and to varying degrees with γ-irradiation.

### γ-FBS does not Support or Enhance the In Vitro Formation of Putative NB/NLP from Human Body Fluids

In a previous study [2], we had noticed that, other than serum, several body fluids obtained from either normal or diseased individuals (e.g. saliva, urine, ascites, cerebrospinal fluid, pleural effusion, and synovial fluid) could produce NB/NLP after incubation for several days or weeks in cell culture conditions or in the presence of supersaturating levels of calcium and phosphate ions. As verified through a proteomic approach, the NB/NLP formed under these conditions were seen to adsorb or “hijack” calcium and apatite binding proteins from the fluid milieu used [2]. Formation of NB/NLP from the various body fluids examined was seen to be largely independent of the need for any feeder serum, contrary to the earlier claims calling for the inclusion of γ-irradiated serum as a requisite condition for this same demonstration [6]–[8], [39], [40]. In our view, this spontaneous formation of NB/NLP seen by us—presumably explained by the aggregation of precipitating ions with surrounding proteins and organic compounds in the fluid milieu—can most likely account for the entire NB phenomenology [1]–[5]. However, it is unclear at this time whether the presence of γ-FBS can indeed enhance the formation of NB/NLP derived from the body fluids inoculated into DMEM, as claimed [6]–[8], [39], [40].

**Figure 11:**
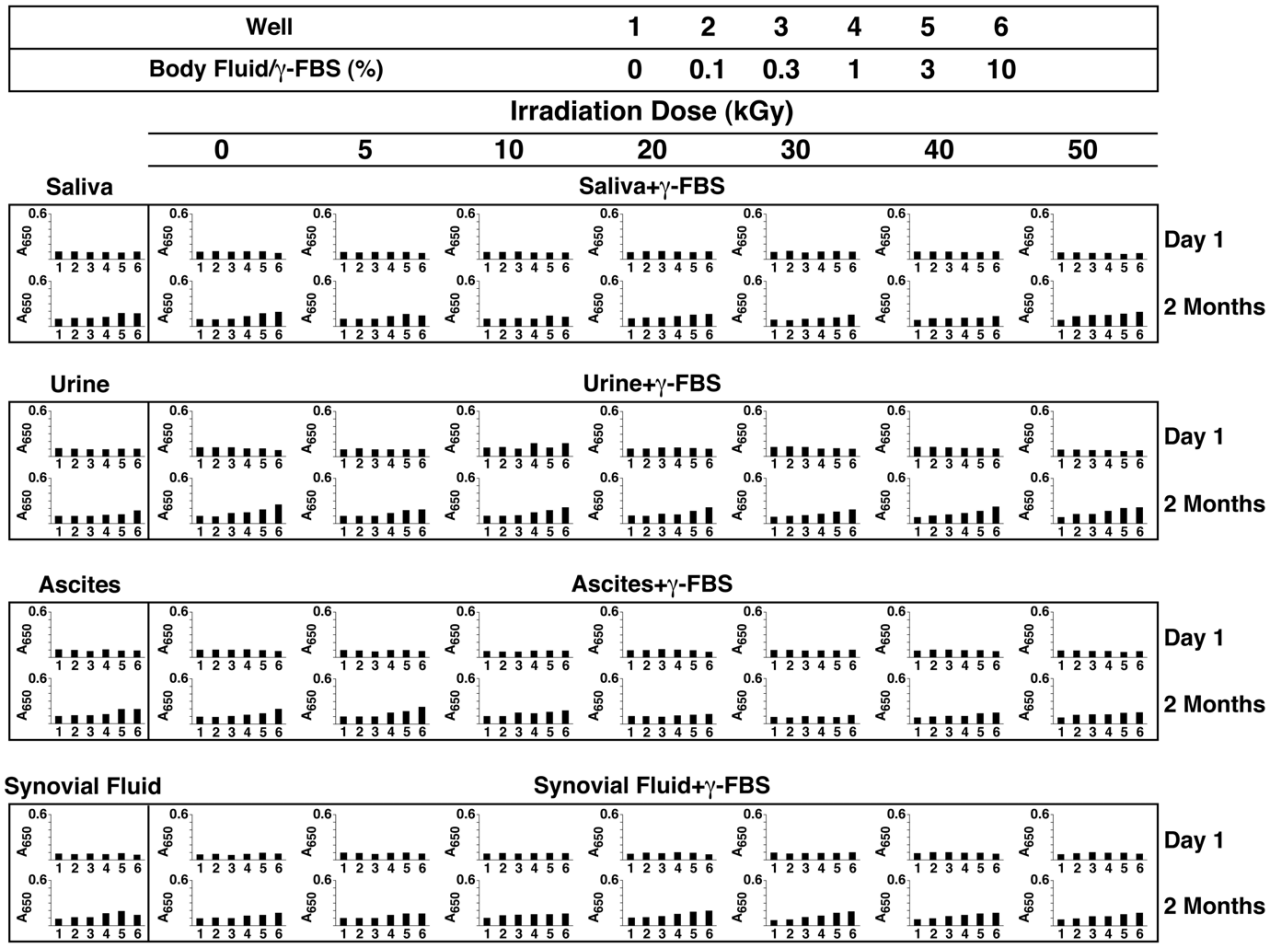
γ-FBS fails to enhance the formation of NB/NLP cultured from human body fluids. Body fluids successively filtered through both 0.2 and 0.1-µm membranes were inoculated into DMEM to the concentrations indicated on the top. In some experiments, non-irradiated FBS (“0 kGy”) or γ-FBS (“5-to-50 kGy”) were added into the DMEM solutions to the same concentrations as the indicated body fluids. A_650_ turbidity readings were performed following inoculation (“Day 1”) or after incubation in cell culture conditions for “2 Months.” Incubation of body fluids, with or without additional FBS/γ-FBS, resulted in similar time-dependent turbidity increases as seen by the straight curves of precipitation seen after 2 months.

In order to verify this contention, we inoculated into DMEM several body fluids at various concentrations and incubated the solutions for several weeks in cell culture conditions (Fig. 11). For these experiments, we used saliva collected from healthy individuals, urine obtained from patients with nephrotic syndrome (i.e. proteinuria), and ascites and synovial fluids collected from patients with various clinical conditions [2]. For brevity, only one set of results for each one of these body fluids is shown in Figure 11. Largely similar results were obtained for all the other body fluid samples tested (not shown). As expected, we noticed slow turbidity increases over a period of two months for all the four body fluids studied (Fig. 11, panels on the left, compare “Day 1” vs. “2 Months” for the various body fluids shown). The body fluids tested produced mostly straight-line turbidity increases as a function of the inoculum sizes. These observations clearly showed that human body fluids could produce NB/NLP irrespective of whether they were obtained from healthy or diseased individuals (see *Materials and Methods* for more sample details).

Surprisingly, the addition of each body fluid in the presence of control, untreated FBS did not result in any additive effect on the formation of NB/NLP when compared to the body fluids incubated alone (Fig. 11, “2 Months,” compare “0 kGy” panels with the panels on the left). About the only minor change detected was a small increase of turbidity seen with the urine sample that had been inoculated into DMEM along with FBS. Likewise, the inoculation into DMEM of both γ-FBS and the various body fluids also did not result in any noticeable enhancement of turbidity increase or precipitation after two months of incubation when compared to the inoculation of the body fluids alone or to the inoculation of these same body fluids in the presence of control FBS (Fig. 11, “2 Months,” compare the “5-to-50 kGy” panels with the panels on the left or with the “0 kGy” panel). These results indicated that γ-FBS did not enhance NB/NLP formation produced by human body fluids under culture conditions.

The absence of feeder effect for γ-FBS was unexpected given that this biological fluid was shown to induce NB/NLP precipitation on its own after 1 month of incubation under the same conditions (Fig. 6A). On the other hand, we also noticed earlier that γ-irradiation appeared to have enhanced the inherent calcification-inhibitory activity of serum in the presence of supersaturating levels of precipitating ions (Fig. 7). It is thus possible that the co-presence of γ-FBS along with body fluids may have enhanced the inhibitory tendencies inherent in γ-FBS at the expense of an opposing propensity towards mineral nucleation. Regardless, given the absence of any feeder effect seen in our experiments, we must conclude that there is no factual basis to justify the use of γ-FBS during the culture of NB/NLP from human samples, contrary to what had been assumed earlier [6]–[8], [39], [40] and that has led to the rampant use of γ-FBS as a feeder in all subsequent studies seeking to demonstrate the presence of both NB and CNP in human tissues (Table 1).

### NB/NLP Cultured from either Untreated Serum or γ-Irradiated Serum Show Similar Morphologies when Observed by Electron Microscopy

In order to confirm that the turbidity increases seen with γ-irradiated serum were actually due to the formation of mineralized structures similar to the earlier described NB/NLP [2]–[4], [6]–[8], we centrifuged the precipitates obtained after incubation of 10% γ-FBS or γ-HS into DMEM for two months (as depicted in Fig. 6) and processed them for electron microscopy (see *Materials and Methods*).

**Figure 12:**
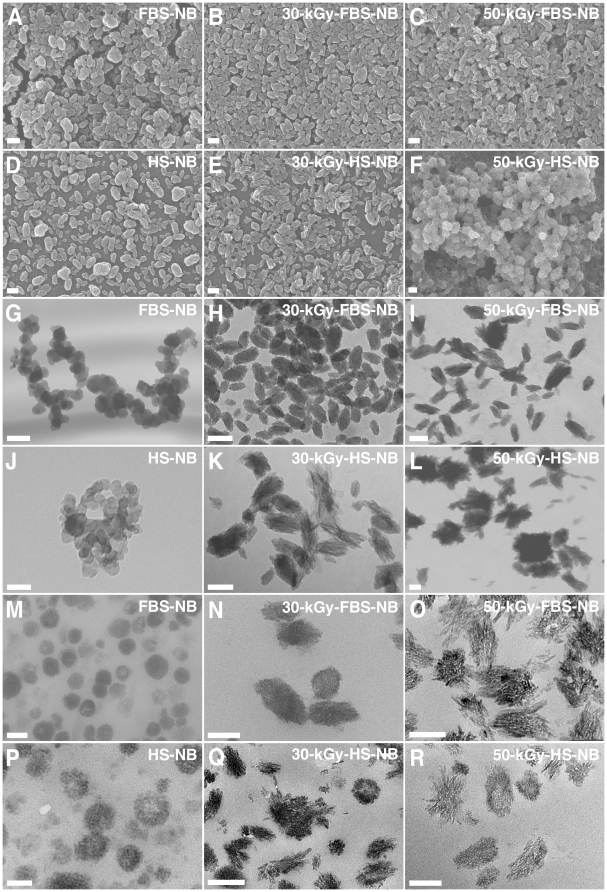
NB cultured from either untreated serum or γ-irradiated serum display similar morphologies under electron microscopy. NB were collected from a 2-month incubation of the indicated serum at 10% as seen in Figure 6. Following centrifugation and washing steps, the NB specimens were processed for (A–F) SEM, (G–L) TEM, or (M–R) thin-section TEM as described in the *Materials and Methods*. NB obtained from either untreated or γ-irradiated serum appeared as small, rounded and elongated particles displaying varying degrees of crystallinity. Scale bars: 50 nm (J, P); 100 nm (I, K, M–O, R); 200 nm (A–E, G, H, L, Q); 500 nm (F).

Under SEM, the NB/NLP specimens obtained from both γ-FBS and γ-HS turned out to be virtually indistinguishable from those obtained from their control, non-irradiated serum counterparts (Fig. 12A–F). That is, round and ellipsoid mineral particles of small sizes with diameters ranging mostly from 50 to 300 nm were seen in all the specimens examined, except for the sample obtained from γ-HS that had been irradiated at 50 kGy (Fig. 12F). At this high γ-irradiation dose and after 2 months of incubation, the NB derived from γ-HS produced round aggregated particles of larger sizes with diameters around 500 nm, an observation which may be attributed to the extensive precipitation of particulate matter observed in this solution prior to incubation (Fig. 1A, HS row, “50 kGy”). With additional incubation, however, all NB specimens were seen to grow in size and to aggregate until they collapsed into films (not shown). These mineral nanoparticles were also morphologically similar to the NB/NLP specimens studied earlier [2]–[4]. In the case of γ-FBS-NB, they were virtually indistinguishable from the control strains of NB that had been cultured from FBS [31] and that had been deposited into the German Collection of Microorganisms and Cell Cultures (DSMZ; Braunschweig, Germany) as strains DSM 5820 (see for example Fig. 3L of ref. [3]). The NB/NLP obtained here were morphologically equivalent to another strain of NB designated as “Nanobacterium sp. strain Seralab 901045” (see Fig. 7K of ref. [2]). This particular NB strain, which was renamed “nanons” by Raoult *et al*. [24], was also initially isolated from FBS [30].

Under TEM, the NB/NLP specimens cultured from both control, untreated sera (FBS and HS) and γ-irradiated sera (γ-FBS and γ-HS) produced a wide spectrum of morphologies (Fig. 12G–L) similar to those described earlier [1]–[5]. Thus, depending on the length of incubation and as demonstrated before [1]–[4], these morphologies included amorphous-looking round nanoparticles and larger ellipsoid particles or platelets, as well as spindles, films, and aggregates showing varying degrees of crystallization, all of which are represented in Figure 12G–L, illustrating again the ability of simple mineralo-organic complexes to duplicate *pleomorphism*, considered earlier as a characteristic and salient morphological feature of NB [6], [12]. In the case of NB/NLP derived from the slow incubation of normal, untreated serum (both FBS and HS), we noticed a higher tendency for the particles to stay rounded, even after a lengthy incubation of up to 2 months (Fig. 12G and J). On the other hand, the NB/NLP derived from γ-irradiated serum (both γ-FBS and γ-HS) showed a higher propensity to convert to more crystalline formations and to include more mature shapes in the form of ellipsoid particles, platelets, spindles, and aggregates (Fig. 12H–I and K–L). However, we have seen a large data scatter here, and, in any given sample, an entire spectrum of morphologies can be identified. Some of these morphologies in the form of elongated particles and spindles were strikingly similar to the prolate ellipsoids referred as secondary “calciprotein particles” or “CPPs” that were derived from the aging and natural conversion of nascent apatite-fetuin-A complexes that initially also assume the form of round particles (termed primary CPPs) [92]–[94]. Similar secondary CPPs had also been isolated earlier from the ascites of a late-stage kidney disease patient suffering from calcifying peritonitis [94]. These elongated, spindle-shaped forms of NB/NLP most likely represent intermediate stages seen during the amorphous-to-crystalline phase conversion of apatite particles, a process that has shown to be irreversible in our hands and that has been seen to progress until there is complete coalescence or collapse of these spindles into films [2]–[5].

When examined by thin-section, the NB samples cultured from both untreated and γ-irradiated sera again showed a wide spectrum of morphologies, ranging from predominantly round particle shapes seen with untreated serum samples (both FBS and HS, Fig. 12M and P) to ellipsoid and spindle-shaped shapes with varying degrees of crystallinity seen more commonly with γ-irradiated sera (both γ-FBS and γ-HS, Fig. 12N and O and Fig. 12Q and R, respectively). Although the round particles appeared to be predominantly amorphous, some samples displayed a certain degree of crystallinity, as illustrated by the HS-NB sample shown in Figure 12P. Again, a variety of shapes and forms could be seen with any given sample and each in fact displayed the entire spectrum of morphologies ranging from amorphous spherical particles to crystalline films.

Taken together, these electron microscopy observations indicate that the mineral complexes cultured from γ-irradiated serum resemble morphologically the NB obtained from the slow culture of medium inoculated with serum [6]–[8] as well as the mineral complexes found in the normal serum [3] or the particles found associated with protein-mineral complexes [4]. In each instance, an entire spectrum of pleomorphic morphologies has been identified that reflects largely the state of amorphous-to-crystalline phase conversion seen with these complexes. In general, at any given incubation time, the particles obtained from γ-irradiated serum appeared more mature than the ones formed from normal serum, but it is not clear whether this difference bears any meaning in terms of the underlying mechanism of particle formation seen under one condition versus the other. Seen from a different perspective, it is clear that the aggregation of apatite complexes can be repressed by various inhibitors (either whole sera or serum proteins, be them irradiated or not), and that the different intermediate morphologies represent various states of de-repression that appear with aging and progressive stages of maturation. All such intermediate forms appear to be transient though and they are expected to yield complete crystalline formations with time.

### NB/NLP Cultured from either Untreated or γ-Irradiated Serum Show Similar Mineral Compositions when Assessed by Energy-Dispersive X-Ray Spectroscopy

In order to confirm that the precipitates cultured from γ-irradiated serum are chemically equivalent to the putative NB described earlier [6]–[8] and to the NLP characterized in our own previous studies [1]–[5], we determined their elemental composition using energy-dispersive X-ray spectroscopy (EDX). The mineral phase of NB was characterized previously as consisting of carbonate HAP [6], [8], [13]–[15], the mineral found in bones and teeth of vertebrates (reviewed in refs. [97], [98]). As such, EDX was in fact used earlier by NB proponents to confirm the presence of NB in various culture specimens [8], [18], [25], [32], [34], [37].

**Figure 13:**
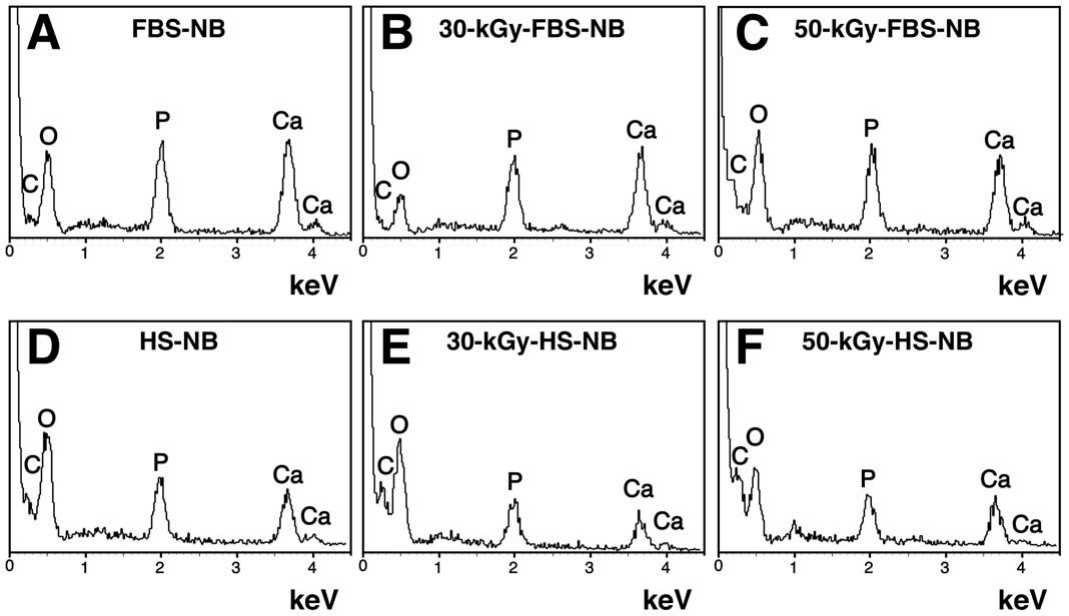
NB cultured from either untreated serum or γ-irradiated serum show similar elemental compositions when analyzed by energy-dispersive X-ray spectroscopy. NB were retrieved from the experiments described in Figure 6. Precipitates were taken from 2-month-old cultures of either untreated FBS (A) or from FBS γ-irradiated at either 30 kGy (B) or 50 kGy (C). In (D–F), NB specimens were obtained from cultures of untreated HS (D) or from HS γ-irradiated at either 30 kGy (E) or 50 kGy (F). Following centrifugation and washing, the specimens were processed for EDX as described in the *Materials and Methods*. Major peaks of carbon (C), oxygen (O), phosphorus (P), and calcium (Ca) were noticed for the various NB specimens, consistent with the presence of a calcium phosphate mineral containing carbonate ions. The following Ca/P ratios were obtained: (A) 1.40; (B) 1.40; (C) 1.24; (D) 0.98; (E) 0.86; and (F) 1.09.

When analyzed using EDX, the NB specimens obtained from both γ-FBS and γ-HS were found to be indistinguishable from those obtained from their control, non-irradiated serum counterparts (Fig. 13A–F). That is, all these specimens showed peaks of carbon, oxygen, phosphorus, and calcium consistent with the presence of carbonate, phosphate, and calcium ions within the mineral phase of NB (Fig. 13A–F). Calcium∶phosphorus ratios varying between 0.86 to 1.40 were obtained for the samples shown, consistent with the range of ratios described earlier for both NB/NLP and carbonate apatite [2]. To confirm the presence of carbonate apatite, we used a combination of FTIR, X-ray diffraction (XRD), and Raman spectroscopy analyses. As expected, we were able to verify that all NB specimens cultured from both untreated and γ-irradiated sera consisted of apatite containing significant amounts of carbonate (data not shown), a result in line with the extensive observations made earlier on NB/NLP samples obtained from both sera and serum proteins [2]–[4].

### Proteomic Analysis Reveals that NB/NLP Cultured in the Presence of both γ-FBS and Human Body Fluids Harbor Both Bovine and Human Proteins

In order to fully understand the role of γ-FBS in the assembly of putative NB, we performed a comprehensive proteomic analysis of the putative NB samples cultured from γ-FBS as well as from the various body fluids supplemented with γ-FBS. We had earlier developed a methodology through which the NB samples obtained from cultures were first treated with 50 mM EDTA, followed by treatment with the protease trypsin in solution, separation of the resulting peptides by liquid-chromatography, and identification by tandem MS/MS analysis (see *Materials and Methods*). The identified proteins were then ranked according to the number of unique peptides detected in each case.

We first verified the effects of γ-irradiation on the bovine proteins found in association with the putative NB obtained through the slow culture of DMEM in the presence of γ-FBS (or FBS), exactly as prescribed by the initial NB proponents [6]–[8], [39], [40]. For these experiments, we chose the two γ-irradiation doses of 30 kGy and 50 kGy, both of which have been shown to induce extensive breakdown of serum proteins. Furthermore, the irradiation dose of 30 kGy is now widely used to obtain a sterilized serum-feeder for the demonstration of NB in human tissues (Table 1, see also refs. [6]–[8], [39], [40]). As seen in Table 7, the composition of proteins found in the NB cultured from γ-FBS largely overlapped with the protein profile obtained for the NB cultured from control, non-irradiated FBS (Table 7, compare the two columns “30-kGy-FBS” and “50-kGy-FBS” with that of “FBS”). For brevity, only a fraction of the proteins identified is shown here. Remarkably, these samples not only appeared to harbor largely the same proteins but they also showed internal consistency in terms of the relative abundance of the proteins identified (Table 7). Given the extensive breakdown of proteins seen in FBS as a result of γ-irradiation at both of the high doses used here (see the SDS-PAGE patterns shown in both Figs. 5 and 8, for example), we were initially surprised to find these marked similarities in protein composition seen between FBS-NB and γ-FBS-NB. For example, among the major proteins found in the FBS-NB and γ-FBS-NB we identified consistently BSA and, to a lesser degree, BSF (Table 7), both deemed to be among the main calcium- and apatite-binding proteins found in the serum [91]–[94], [99]–[105]. In addition, several other proteins identified here in both FBS-NB and γ-FBS-NB (Table 7) seemed to overlap with those found in an earlier proteomic study of FBS-NB (see Table 1 of ref. [3]), and these proteins included α-1-antiproteinase, apolipoprotein A1, complement component 3, prothrombin, and hemoglobin fetal subunit β (Table 7). In all instances, the proteins identified in both FBS-NB and γ-FBS-NB were virtually identical.

**Table 7:** Proteins of NB/NLP derived from γ-FBS and human urine identified by liquid-chromatography-MS/MS analysis.

			NB Cultured from:						
#	Identified Protein	MW** (kDa)	FBS	30-kGy-FBS	50-kGy-FBS	Urine	FBS+Urine	30-kGy-FBS+ Urine	50-kGy-FBS+ Urine
1	Bovine serum albumin	69	58	61	54	–	48	53	55
2	Bovine serotransferrin	78	28	33	31	–	24	29	29
3	Human serum albumin	69	–	–	–	29	25	35	35
4	Bovine α-1-antiproteinase	46	16	19	19	–	17	16	18
5	Bovine α-fetoprotein	69	16	17	16	–	12	12	14
6	Bovine fetuin-A	38	13	14	14	–	10	13	13
7	Bovine apolipoprotein A1	30	14	15	15	–	10	10	11
8	Bovine complement component 3	187	12	16	15	–	6	5	11
9	Bovine vitamin D-binding protein	53	11	14	12	–	5	7	7
10	Bovine prothrombin	71	9	10	9	–	3	9	8
11	Bovine hemoglobin fetal subunit β	16	8	8	8	–	6	8	10
12	Bovine α-1B-glycoprotein	54	3	11	9	–	3	8	8
13	Human complement component 3	187	–	–	–	35	0	0	0
14	Bovine inter-α-trypsin inhibitor H4	102	7	9	7	–	0	5	6
15	Bovine α-1-acid glycoprotein	23	6	7	6	–	3	5	6
16	Bovine serpin A3-1	46	4	7	7	–	0	5	7
17	Human serotransferrin	77	–	–	–	30	0	0	0
18	Human α-1-microglobulin/bikunin	39	–	–	–	8	6	8	7
19	Bovine fetuin-B	43	3	6	7	–	2	4	4
20	Bovine α-2-antiplasmin	55	5	5	4	–	2	3	3
21	Bovine plasminogen	91	5	6	5	–	0	2	3
22	Bovine kininogen-1	69	2	7	4	–	0	4	3
23	Bovine α-1-microglobulin/bikunin	39	4	5	6	–	0	2	3
24	Human α-2-macroglobulin	163	–	–	–	9	4	3	4
25	Human α-1-acid glycoprotein 1	24	–	–	–	7	3	4	5
26	Bovine PEDF*	46	2	7	3	–	0	3	3
27	Bovine β-2-glycoprotein 1	38	3	3	4	–	0	2	3
28	Bovine hemopexin	52	3	5	5	–	0	0	2
29	Bovine factor XIIa	52	2	4	2	–	0	2	3
30	Bovine apolipoprotein A2	11	2	3	2	–	0	2	3
31	Human α-1-antitrypsin	47	–	–	–	12	0	0	0
32	Human inter-α-trypsin inhibitor H2	106	–	–	–	2	2	3	4
33	Human Ig κ chain C region	12	–	–	–	4	2	2	2
34	Human Ig λ chain C region	11	–	–	–	4	2	2	2
35	Human Ig γ-1 chain C region	36	–	–	–	9	0	0	0
36	Human apolipoprotein A1	31	–	–	–	8	0	0	0
37	Human Ig α-1 chain C region	38	–	–	–	8	0	0	0
38	Human haptoglobin	45	–	–	–	7	0	0	0
39	Bovine antithrombin-III	52	3	0	0	–	2	0	0
40	Human apolipoprotein B100	516	–	–	–	1	0	0	0

*PEDF: Pigment epithelium-derived factor.

**MW: molecular weight.

The value shown in each case corresponds to the number of unique peptides identified as described in the *Materials and Methods*. All samples were prepared from culturing 10% of the corresponding serum and/or urine for two months in cell culture conditions.

These results were all the more surprising since the entire rationale for using γ-FBS as a feeder to support NB cultures was founded on the presumption that the γ-FBS played no role in the assembly of NB! In fact, our data demonstrated clearly that γ-FBS not only induced by itself the formation of NB but it also contributed directly to the protein composition of NB in a manner that was virtually indistinguishable from that seen with control, untreated FBS. These unexpected results suggest in turn the possibility that, despite its use as a feeder, γ-FBS may in fact be contributing directly to defining the protein composition of the NB “grown” in its presence, a bizarre scenario that we next seek to confirm further.

Using the procedure established for Figure 11, we first cultured various body fluids in DMEM in the absence of any serum in order to derive NB which were then subjected to the same proteomic analysis. The results for one series of experiments done with the urine of a patient with proteinuria are shown here. That is, we derived urine-NB by culturing urine in serum-free DMEM. The proteomic analysis of this material revealed several proteins found also in the human serum and known to bind to calcium or apatite, including HSA, complement component 3, serotransferrin, α-1-microglobulin/bikunin, α-2-macroglobulin, α-1-acid glycoprotein 1, α-1-antitrypsin, inter-α-trypsin inhibitor H2, haptoglobin, as well as several apolipoproteins and immunoglobulins (Table 7, “Urine” column). When we compared the protein profiles of urine-NB with those of NB cultured in the presence of both FBS and urine (“FBS+Urine”), we noticed that, with the exception of HSA, the other major proteins initially found in the urine-NB were either absent or found in drastically reduced frequency in FBS-urine-NB (Table 7). Thus, while human complement component 3, serotransferrin, apolipoproteins A1 and B100, α-1-antitrypsin, haptoglobin, and various immunoglobulins (Ig) were present in relatively high amounts in urine-NB, these proteins were not detected when FBS was also present in culture (Table 7, “FBS+Urine” column). Similarly, the number of unique peptides corresponding to the human proteins α-2-macroglobulin, α-1-acid glycoprotein 1, Ig κ chain C region, and Ig λ chain C region were found to decrease in the presence of FBS (Table 7). Instead of the human proteins initially found in urine-NB, we detected an entire repertoire of bovine serum proteins associated with the scaffold of FBS-urine-NB that largely mirrored the protein profile seen earlier with FBS-NB (Table 7, compare the “FBS+Urine” column with that of “FBS”). In fact, with the exception of HS and a few minor human proteins, it appears that the bovine serum proteins largely dominated the composition of proteins found in the FBS-urine-NB scaffold, a result which in our view may have simply reflected a much higher protein concentration associated with FBS compared with that found in the human urine used here. Accordingly, we observed that the total protein concentration of the non-irradiated FBS used was around 32 times higher than that of the urine obtained from the proteinuria patient (i.e. 32 mg/ml for FBS vs. 1 mg/ml for the urine used; see ref. [4] and *Materials and Methods*).

A similar protein composition was found when NB were cultured from both urine and γ-FBS irradiated at either 30 or 50 kGy (Table 7, “30-kGy-FBS+Urine” and “50-kGy-FBS+Urine”). That is, there was a noticeable decrease in the frequency of the human proteins found earlier with urine-NB, and, again, with the exception of HSA, the main proteins found in the NB scaffolds here appeared to consist of bovine serum proteins. In fact, with the exception of HSA and a few other minor human proteins, the protein profiles of both 30-kGy-FBS-urine-NB and 50-kGy-FBS-urine-NB were virtually indistinguishable from their γ-FBS-NB counterparts (Table 7). The predominance of bovine proteins over human proteins seen here appears to reflect again disparities in the respective protein concentrations found for these fluids. Thus, the total protein concentrations of the γ-FBS samples used were 26 mg/ml for γ-FBS (30 kGy) and 23 mg/ml for γ-FBS (50 kGy) vs. 1 mg/ml for the urine.

Similar results were obtained with the other body fluids cultured in the presence of γ-FBS. That is, the resulting NB were all shown to contain bovine serum proteins in addition to the human fluid proteins (data not shown). The predominance of any given set or species of proteins in the final composition of NB (i.e. bovine proteins found in γ-FBS or human proteins from the body fluid) appeared to be mainly a function of the protein concentrations associated with the fluids used to generate NB (i.e. γ-FBS vs. body fluid, data not shown).

These findings were unexpected since we had assumed that the proteins from γ-FBS would have been ionized and fragmented to the point that they would have been rendered unrecognizable when examined by proteomics. This clearly was not the case since the protein profiles of NB obtained in the presence of γ-FBS were virtually indistinguishable from those obtained from untreated FBS. Evidently, enough proteins or protein fragments were present in γ-FBS to propitiate their avid binding to the nascent apatite complexes that then result in the formation of the particles described as NB.

The results presented here demonstrate another peculiar aspect of the NB biology. The rampant use of γ-FBS as a feeder for NB cultures may have resulted in the introduction of *bovine* proteins into the composition of a supposedly *human* pathogen! This unprecedented scenario raises serious concerns regarding the many studies linking NB in human tissues to various disease processes (Table 1). In our view, our results nullify all these previous findings made with γ-FBS. Our results call into question even the more recent studies done on the so called CNP (Table 1). Here, CNP obtained from human tissues were inoculated into laboratory animals and cell cultures in order to establish a pathological role for these particles [30], [34], [38], [52], [53]. Intriguingly, γ-FBS was also used as a feeder to generate the CNP in question [30], [34], [38], [52], [53]. Needless to say, these same CNP must have also carried a hybrid combination of proteins originating from both the γ-FBS and the human tissue being studied. Upon inoculation into rabbits, the CNP may very well have acquired additional antigens derived from a yet another (i.e. third) species [34], [38], [53]! This bizarre scenario makes it difficult, if not impossible, to interpret the pathology data obtained in those studies. In fact, it is not even clear which proteins (human or bovine, or perhaps a third species corresponding to the host) are causing the putative tissue damage being recorded.

### Conclusion and Future Perspectives

To date, the rampant use of γ-irradiated serum has led to the publication of a vast body of literature implicating NB in the pathogenesis of numerous diseases (Table 1). All these studies were based on the false assumption that the γ-irradiated serum, being sterilized and rid of the presence of any residual NB in the serum, would provide an excellent feeder for the subsequent culture of NB or NB precursors derived from human and animal tissues [6]–[8], [39], [40]. The results presented here demonstrate however that all the previous studies based on the use of γ-irradiated sera as feeder and as listed in Table 1 are fundamentally flawed. This conclusion stems from the following considerations.

Rather than being “sterilized” or rid from the influence of any residual NB activity in the serum, both γ-FBS and γ-HS are shown here to be able to produce NB/NLP when incubated in cell culture conditions in a manner similar to normal, non-irradiated serum. In fact, the γ-irradiated serum is shown to produce actively NB/NLP through a dual mechanism of calcification inhibition and seeding, exactly as seen also with normal, untreated serum. Moreover, γ-FBS does not in fact support or enhance the formation of NB/NLP from human body fluids, as it had been deemed (or assumed) to do. As such, there is no basis whatsoever to support the use of γ-FBS as a feeder for the slow growth of NB or NB-related particles like CNP in culture. It is also clear that γ-FBS contributes actively to the formation of NB/NLP through the protein fragments still present in the serum in spite of the strong irradiation used. In fact, the protein composition of NB/NLP formed in the presence of either γ-FBS or γ-HS resembles closely that identified for NB/NLP formed from normal, untreated serum. From a mechanistic point-of-view, both the γ-irradiated sera and the γ-irradiated proteins are shown to exert the dual effects of inhibition and seeding on NB/NLP formation in a manner virtually indistinguishable compared with their normal, untreated counterparts. This is a surprising finding since extensive serum protein breakdown is seen with γ-irradiation at the doses of irradiation previously used by the NB/CNP proponents. It is thus clear that all so-called NB/CNP derived from human tissues and cultured in the presence of γ-FBS actually harbor antigens derived from γ-FBS in addition to proteins derived from the human tissue under investigation. In other words, the NB that are presumed to be derived from human tissues are in fact being assembled from the combined influence of both the tissue under investigation and the “feeder” that supposedly serves as a source of nutrients! This latter observation, demonstrated through the rigorous application of a comprehensive proteomics approach, can be seen to explain a rather unprecedented, if not confusing, scenario in which particles that are claimed to be pathogenic in the context of a human disease are actually comprised of proteins and organic entities derived from both human and bovine origins! By itself, this erroneous and highly misleading representation should provide sufficient ground to nullify the entire body of literature accumulated on NB to date.

It is true that more recent studies on NB have indeed acknowledged the controversial nature of the NB phenomenology and the possibility that NB are not alive at all—resulting in the coining of the term “calcifying nanoparticles” or CNP to replace the so-called NB [14], [20], [38], [51]–[53], [106]. However, it is equally intriguing that all these later studies have continued to use γ-FBS as a “feeder” to support the growth and propagation of CNP from normal and diseased tissues [38], [51]–[53]. In fact, the successful production of CNP in cultures containing γ-FBS as feeder has been used as definitive proof in favor of the NB hypothesis. More surprisingly, these same CNP, harboring hybrid proteins from both human and bovine origins, have in turn been inoculated into laboratory animals (i.e. rabbits and rats) for the demonstration of various disease processes that include vascular calcification seen in atherosclerotic lesions and formation of gallstones [34], [38], [53], [107]. These same animal experiments have since become part of a broader claim as having fulfilled successfully, or at least in part, Koch's postulates, long used in the past for the validation of classic infectious disease processes [15], [20], [34], [38], [107]–[109]. Based on these studies, CNP have continued to be deemed pathogenic even though they are no longer claimed to be alive [38], [53], [106], just as NB were deemed “infectious” and “contagious” earlier [7]–[9], [11], [13], [15], [17], [109]. Our results presented here indicate strongly that all such claims are questionable, if not flawed. Even more alarmingly, our data point to the insurmountable dilemma that, most likely, the hybrid CNP containing proteins and other organic moieties originating from two species (i.e. human and bovine) may very well have ended up acquiring antigens and organic moieties from a third species (i.e. host animal inoculated with CNP, be it rabbit, rat, or some other animal), and, yet, such a confusing scenario is being used to support claims of pathogenicity for the inoculated host species, and, by extension, with implications to human pathogenesis!

Our studies would even question the validity of any conclusions reached earlier with cell lines as targets of NB [30]. This study had indicated a cytotoxic effect of NB on various cell lines. Based on our data shown here, it is questionable whether NB/NLP structures carrying hybrid proteins (human and bovine antigens), shown to be cytotoxic to a yet third cell species, can have any meaningful or physiological significance!

It is not clear to us why earlier studies using γ-FBS as a feeder for NB cultures did not detect spontaneous seeding associated with this irradiated serum inoculated into culture medium [6]–[8], [39], [40]. One possibility is that both normal and γ-irradiated serum produce NB-like precipitates only upon long incubations that normally take several weeks to develop. In other words, like its normal serum counterpart, the γ-irradiated serum is able to seed particles only slowly, following several weeks of incubation, and this lengthy process may have produced spurious and erratic seeding patterns that have gone unnoticed before.

Until this present study, the only remaining body of evidence related to NB that had not yet been refuted by us [1]–[5] and others [21]–[24] pertained to the widespread use of γ-irradiated serum as a feeder for the growth and demonstration of both NB and CNP (Table 1). By invalidating any justification for the use of γ-irradiated serum as a feeder in the context of both NB and CNP, our results now impel us to conclude definitively that there is in fact no real evidence that can be seen to validate any of the NB/CNP phenomenology, particularly any relationship between NB/CNP and disease processes, at least in the manner that it had been claimed.

While disproving the NB hypothesis, our own results demonstrate that NLP as biological entities are in fact widely distributed in nature and are ubiquitously present in all body fluids [1]–[5]. Our results point however to a much simpler explanation for their biology than that advanced through the NB literature. According to our model, NLP are normal constituents of the broader calcium homeostasis seen throughout nature. In fact, in our view, NLP are used as part of the normal pathways of osmoregulation, storage, mobilization, excretion, detoxification, and waste disposal of ions and minerals [2]. More precisely, NLP can be seen as physiological remnants of normal calcium homeostasis that accumulate at times when there is an excess of precipitating ions [2]. In fact, mineral-protein and mineral-organic complexes resembling NLP have been described in the past as precursors in the formation of bones and teeth in vertebrates [110]–[113]. From a broader perspective, NLP can be seen as part of an even more general family of similar mineral-organic complexes, which we have termed *bions* [5], that biomimetically resemble complex biological structures and shapes, and that are chemically defined by entities undergoing various stages of amorphous-to-crystalline transformation.

It remains to be seen whether structures resembling NB/NLP/CNP, now known to be present in the normal serum [3], can result in disease processes. In our view, while these entities must be seen as innocuous constituents of the physiological cycle regulating normal calcium homeostasis, any overt accumulation of such complexes, for instance, along vascular walls or as kidney deposits, can propitiate a disease cycle of its own [5]. In fact, this contention is probably true with just about any metabolite in the body that overtly accumulates under certain pathophysiological conditions.

In support of this view, it should be mentioned that protein-mineral nanoparticles resembling the NB/NLP described in this study have been found in pathological conditions including calcifying peritonitis [94], [114] and ectopic calcifications induced by drugs which disrupt calcium homeostasis (i.e. vitamin D, warfarin, etidronate, and adenine) [115]–[119]. It is possible that the NB/NLP may also be found in other conditions including aging, kidney failure, diabetes, hyperparathyroidism, inflammation, and atherosclerosis, all of which are associated with ectopic calcifications [120]–[124].

Contrary however to the untenable claims made earlier for both NB and CNP, we contend that any disease claims must be predicated first on a clear elucidation of the molecular and chemical structure of the candidate disease agent, in this case NB/NLP/CNP, which we believe to have unraveled through this and previous studies [1]–[5]. We also believe that any putative candidate for pathogenesis must first attend to the rules governing normal biological cycles, as opposed to the premature and perhaps extraneous need to fulfill claims for exotic and microbiologically unprecedented characteristics as called earlier by both NB and CNP proponents. With a more thorough characterization of these mineralo-organic complexes as seen here and elsewhere [1]–[5], it is now possible to demystify their biology as it unfolds within the body and throughout nature. Attending also to these commonsensical considerations, we are now finally in a position to assess the potential roles of these entities in health and disease.

## Materials and Methods

### γ-Irradiation of Serum and Protein Solutions

Approval for the use of human samples was granted by the Institutional Review Board of Chang Gung Memorial Hospital (Gueishan, Taoyuan, Taiwan). Written informed consents were obtained from the individuals who provided the samples. Human blood was collected from healthy human volunteers using a conventional venipuncture technique after sterilization of the skin with ethanol. Whole blood was collected into sterile serum collection tubes containing no anticoagulant (Becton, Dickinson & Company, Sparks, MD, USA). The blood was left to coagulate for 30 min at room temperature without agitation. The coagulated blood was centrifuged at 1,500×*g* for 15 min at room temperature. The supernatant corresponding to HS was sterilized by successive filtration through both 0.2-µm and 0.1-µm membranes (Pall Corporation, Ann Arbor, MI, USA), and was stored at −20°C until use. Commercial FBS (Biological Industries, Kibbutz Beit Haemek, Israel) was incubated in a water bath at 56°C for 30 min to inactivate complement proteins. The resulting complement-inactivated FBS was sterilized by filtration as described above, and was stored at −20°C until use.

For γ-irradiation, 140 ml of filtered HS or filtered FBS was separated into seven fractions of 20 ml each. The solutions were placed into sterile, 100-ml glass bottles (Schott, Hattenbergstrasse, Germany). Each bottle was subjected to γ-irradiation treatment at a dose ranging from 5 to 50 kGy. γ-Irradiation was performed in accordance with local regulations (Institute of Nuclear Energy Research, Atomic Energy Council, Lungtan, Taoyuan, Taiwan) using radioactive cobalt-60 (^60^Co) as a source of γ-rays. The dose of γ-irradiation for each treatment was monitored using a radiachromic dosimeter film (FWT-60; Far West Technology, Goleta, CA, USA). Following γ-irradiation, the sera were kept at −20°C until use.

For the γ-irradiation of serum proteins, lyophilized BSA (Sigma, St-Louis, MO, USA), BSF (AppliChem, Boca Raton, FL, USA), or HSA (Sigma) were dissolved individually at a final concentration of 10 mg/ml into either double-distilled water or HEPES buffer (20 mM HEPES, 1 mM CaCl_2_, 2 mM Na_2_HPO_4_, and 150 mM NaCl, pH 7.4). The protein solutions were sterilized by 0.2-µm-membrane filtration, and they were transferred into 100-ml sterile glass bottles for γ-irradiation at either 5 or 30 kGy. Following γ-irradiation, the protein solutions were stored at −20°C until use.

### Spectrophotometry and Protein Fluorometry

Visual inspection, photography, and turbidity reading of solutions in 24-well plates (Corning, Corning, NY, USA) were performed as described earlier [2]. Unless otherwise indicated, photography and turbidity reading referred as “Day 1” were performed within one hour following preparation of each 24-well plate. To monitor the absorbance of UV and visible light by the serum and protein solutions, 1 ml of each solution was transferred into a disposable, transparent, quartz or plastic cuvette, and absorbance was monitored in scanning mode at wavelengths spanning from 200 to 750 nm.

For protein fluorescence, 1 ml of serum or protein solutions was transferred into a quartz cuvette. Fluorescence emission was measured at wavelengths ranging from 300 to 450 nm following excitation at 280 nm using a Cary Eclipse fluorescence spectrophotometer (Varian, Palo Alto, CA, USA).

### Fourier-Transformed Infrared Spectroscopy

For FTIR analysis, a 2-µl aliquot of serum or protein solutions was placed between two disks of solid KBr, and FTIR spectra were recorded from 4,000 to 800 cm^−1^ using a FT-730 FTIR spectrophotometer (Horiba, Tokyo, Japan) as described earlier [2]. The FTIR analysis was performed essentially as described [79]. Briefly, the baseline of each FTIR spectrum shown in Figure 4 was aligned at 4,000 cm^−1^. For the peak-deconvolution analysis, amide-I peaks were first normalized from 1,700 to 1,600 cm^−1^ using the Gaussian-Lorentzian (GL) function of the Plot program (version 0.997; Apple, Cupertino, CA, USA), and the resulting peaks were shown in the insets of Figure 4. The 4^th^ derivative of each spectrum was obtained by using the differentiation function of the Plot program. The area under the curve for each peak was then determined using the Plot program. The peaks corresponding to α-helix (1,650–1,657 cm^−1^), β-sheet (1,612–1,640 cm^−1^; 1,626–1,640 cm^−1^), β-turn (1,655–1,675 cm^−1^; 1,680–1,696 cm^−1^), and random coil (1,640–1,651 cm^−1^) were selected based on a previous study [125].

### Sodium Dodecyl Sulfate-Polyacrylamide Gel Electrophoresis

Proteins from serum and protein solutions were separated by gel electrophoresis in denatured and reducing conditions as described [2]. For the gels shown in Figure 5A and B, 0.3 µl of each serum sample was dissolved in water to obtain a final volume of 16 µl. For Figure 5C and D, 2 µl of each protein solution (all at 10 mg/ml) was dissolved in water to a final volume of 16 µl. Each sample was mixed with 4 µl of the 5X “loading buffer” (0.313 M Tris-HCl, pH 6.8, 10% SDS, 0.05% bromophenol blue, 50% glycerol, 12.5% β-mercaptoethanol) to obtain a final concentration of “loading buffer” of 1X and a final loading volume of 20 µl. Every protein solution was heated at 95°C for 5 min prior to loading onto a 10% SDS-polyacrylamide gel. The gels were stained with Coomassie blue and photographed using a black-and-white camera as described earlier [2].

For the gels shown in Figure 8, NLP were prepared by first diluting each serum at a final concentration of 10% into DMEM (Gibco, Carlsbad, CA, USA). Aliquots of 0.25 M CaCl_2_ and 0.25 M NaH_2_PO_4_ (both adjusted to pH 7.4) were then added into each solution to obtain a final concentration of 3 mM each [2]. A final volume of 1 ml of DMEM was used for these experiments. Incubation of NLP was performed with end-to-end agitation for 2 hours at room temperature. NLP were then pelleted by centrifugation at 16,000×*g* for 15 min at room temperature. The NLP pellets were washed twice with HEPES buffer using the same centrifugation steps. The particles were resuspended in 50 µl of 50 mM EDTA. Twelve-µl aliquots of NLP suspensions prepared in FBS (Fig. 8A) or 2 µl of their HS counterparts (Fig. 8B) were mixed with 50 mM EDTA to obtain a final volume of 16 µl. Four µl of the 5X “loading buffer” was added to each sample to obtain a final concentration of “loading buffer” of 1X. The protein solutions were then processed as described above for gel electrophoresis.

### Culture of NB/NLP from γ-Irradiated Serum and Body Fluids

NB/NLP were cultured from FBS or HS as described before [8]. Briefly, non-irradiated serum or γ-irradiated serum was diluted into DMEM to final concentrations ranging from 0.1 to 10%. Twenty-four-well plates were used as described [2], [3]. The plates were incubated at 37°C for at least 2 months in the humidified atmosphere of a cell culture incubator.

For the culture of NB/NLP from body fluids, human saliva was collected from fasting healthy volunteers. One part of protease inhibitor cocktail (Sigma) was mixed with 100 parts of saliva, and the solution was kept at −20°C until use. Other human body fluids, including urine from patients with nephrotic syndrome and showing signs of proteinuria, as well as ascites and synovial fluid from individuals with various clinical conditions, were described previously [2]. For each type of body fluid tested, experiments were performed with samples obtained from five different individuals. Culture of NB/NLP from these body fluids was performed following successive filtration through both 0.2 and 0.1-µm membranes. Filtrated body fluids were diluted into DMEM to final concentrations ranging from 0.1 to 10% using a final volume of 1 ml. In some experiments, non-irradiated FBS or γ-FBS was added to final concentrations ranging from 0.1 to 10% into the DMEM containing body fluids. Culture was performed in triplicates.

### Inhibition of NLP Formation by γ-Irradiated Serum and γ-Irradiated Fetuin-A/Albumin

For the experiments shown in Figure 7, the precipitating reagents CaCl_2_ and NaH_2_PO_4_ were added to a final concentration of 3 mM each to DMEM containing either non-irradiated serum or γ-irradiated serum at concentrations ranging from 0.1 to 10%. A final volume of 1 ml of DMEM was used. The 24-well plates were incubated in cell culture conditions for 2 weeks. Following incubation, photography was taken and turbidity reading was monitored as described above.

For the experiments depicted in Figure 10, the precipitating reagents CaCl_2_ and NaH_2_PO_4_ were added to a final concentration of 3 mM each into DMEM containing either non-irradiated or γ-irradiated forms of BSF and/or BSA at concentrations ranging from 0.02 to 4 mg/ml. A final volume of 1 ml of DMEM was used. In this case, the 24-well plates were incubated in cell culture conditions for either 1 hour (“Day 1”) or 3 days. The plates were processed for photography and turbidity reading as described above.

### Seeding of NLP by γ-Irradiated Fetuin-A and γ-Irradiated Albumin in Metastable Medium Versus Medium Containing Submillimolar Amounts of Calcium and Phosphate Ions

For the experiments shown in Figure 9, solutions of BSF and BSA prepared in HEPES buffer were diluted into DMEM, individually or in combination, to concentrations varying from 0.02 to 4 mg/ml. In some experiments, the precipitating reagents CaCl_2_ and NaH_2_PO_4_ were added to final concentrations of either 0.3 or 0.5 mM each. A final volume of 1 ml was used. In these experiments, the 24-well plates were incubated in cell culture conditions for either 1 hour (“Day 1”) or 1 month. Photography was taken and turbidity reading was monitored as described above.

### Scanning and Transmission Electron Microscopies

NB specimens cultured from either non-irradiated serum or γ-irradiated serum (each at 10%) in DMEM were centrifuged at 16,000×*g* for 15 min at room temperature. NB samples incubated for a period of 2 months were used for Figure 12. The pellets were washed twice with HEPES buffer using the same centrifugation steps. The particles were resuspended in 50 µl of water, and the solutions were used for the various microscopy analyses. For SEM, washed particles were deposited onto formvar, carbon-coated grids (Electron Microscopy Sciences, Fort Washington, PA, USA). The grids were incubated for 5 min at room temperature, and the excess liquid was removed with an absorbent paper. The grids were dried overnight under a laminar flow hood. Prior to SEM observation, each specimen was coated with gold for 90 sec. SEM observations were performed with an S-5000 field-emission SEM (Hitachi Science Systems, Tokyo, Japan).

For TEM, washed specimens were deposited onto formvar, carbon-coated grids, and were dried overnight as described above. For thin-sections, washed particles were dehydrated with two washes of 100% ethanol using the same centrifugation steps described above. The samples were embedded in 200 µl of Epon 812 resin (Electron Microscopy Sciences) with gentle, end-to-end agitation overnight at room temperature. The samples were pelleted by centrifugation at 16,000×*g* for 15 min, and the resin was allowed to polymerize by incubating at 72°C for 2 days. Thin-sections were prepared using a Leica Ultracut UCT microtome (Leica Microsystems GmbH, Wetzlar, Germany). All TEM samples were prepared without additional fixative or staining reagents. TEM observations were performed using a JEOL JEM-1230 electron microscope (JEOL, Tokyo, Japan) operated at 120 keV.

### Energy-Dispersive X-Ray Spectroscopy

Washed and dried NB/NLP were prepared as for SEM analysis but without gold coating. The samples were observed using an S-3000N SEM (Hitachi Science Systems) equipped with an EMAX Energy EX-400 EDX device (Horiba). The EDX analysis was performed in triplicates as described previously [2].

### Proteomic Analysis

In-solution trypsin digestion and high performance liquid chromatography-MS/MS analysis were performed as described earlier [2], [3]. Briefly, NB/NLP were prepared from culturing 10% of either FBS and/or urine from proteinuria patients for 2 months in cell culture conditions as shown in Figure 11. The samples were prepared as described above for microscopy analyses. The washed pellets were resuspended in 50 mM EDTA, and were successively reduced with dithiothreitol, alkylated with iodoacetamide, and digested with trypsin [2], [3]. Dried, trypsinated peptides were mixed with 0.1% formic acid, loaded onto a reverse-phase, liquid chromatography column (Zorbax 300SB-C18, Agilent Technologies, Wilmington, DE, USA), and separated on a 10-cm analytical C18 column (New Objective, Woburn, MA, USA). Peptides eluted from the column were ionized with a 2-D linear ion trap mass spectrometer (LTQ-Orbitrap; Thermo Fisher Scientific, Waltham, MA, USA). Mass fingerprints search and criteria for positive protein identification were done essentially as described [2], [3].

### Protein Quantification

Protein quantification was performed using Bradford assay as described [4]. Optical density was monitored at a wavelength of 595 nm using a spectrophotometer (Molecular Devices, Sunnyvale, CA, USA). The protein concentrations mentioned in the text represent averages of determinations performed in triplicates.
